# A Virus‐Inducible E3–RLCK–MADS Module Coordinates Suppression of Plant Immunity and Fertility in Rice

**DOI:** 10.1002/advs.75503

**Published:** 2026-05-03

**Authors:** Yuansheng Wu, Fengling Wu, Shiting Huang, Denglu Yang, Xin Lei, Xue Zhang, Na Liu, Peng Gan, Chuan Li, Jie Zhang, Ming Wu, Qingxiao Jia, Shanshan Zhao, Jianguo Wu

**Affiliations:** ^1^ State Key Laboratory of Agriculture and Forestry Biosecurity Center For Genetic Improvement Vector‐borne Virus Research Center College of Plant Protection Fujian Agriculture and Forestry University Fuzhou China; ^2^ Institute of Biotechnology and Germplasm Resources Yunnan Provincial Key Lab of Agricultural Biotechnology Yunnan Academy of Agricultural Sciences Kunming China

**Keywords:** MADS1/15, P3IP1, rice, rice grassy stunt virus, RLCK22

## Abstract

Viruses often hijack host developmental programs to promote infection, but the mechanistic links between reproductive regulation and antiviral immunity remain incompletely understood. Here, we identify a virus‐triggered hierarchical degradation cascade that links antiviral immunity and fertility regulation in rice. We show that the rice grassy stunt virus (RGSV) effector P3 transcriptionally activates P3IP1, a RING‐type E3 ubiquitin ligase. P3IP1 ubiquitinates and destabilizes the receptor‐like cytoplasmic kinase RLCK22, which functions as a scaffold to stabilize the floral MADS‐box transcription factors MADS1 and MADS15. The loss of RLCK22 results in decreased MADS1/15 protein levels, accompanied by reduced pollen viability and increased susceptibility to viral infection. Genetic and biochemical analyses support the existence of a regulatory module involving P3IP1, RLCK22, and MADS1/15. Mutants of *mads1*, *mads15*, or *rlck22* exhibit overlapping molecular and antiviral phenotypes, including altered pollen viability and impaired transcriptional responses to RGSV. Our findings uncover a virus‐inducible E3–RLCK–MADS axis linking post‐translational regulation of development and defense, providing new insight into how pathogens manipulate plant fitness through targeted protein degradation.

## 1 Introduction

Balancing reproductive development and pathogen defense is a fundamental challenge for plants [1, 2]. The mechanisms by which flowering plants adjust their development to withstand pathogen invasion are still under investigation. Accumulating evidence suggests that transcription factors, kinases, and post‐translational regulators controlling development are often repurposed during immune responses, but the mechanistic coordination of these processes remains largely unexplored [3, 4, 5, 6, 7].

Among these regulators, receptor‐like cytoplasmic kinases (RLCKs) have emerged as critical signaling nodes that integrate immune perception and developmental control [8, 9, 10, 11]. In *Arabidopsis*, RLCK‐VII members such as BOTRYTIS‐INDUCED KINASE 1 (BIK1), PBS1‐LIKE34 (PBL34), and PBL35 play essential roles in pattern‐triggered immunity (PTI) by transmitting signals from surface‐localized receptors to downstream mitogen‐activated protein kinase (MAPK) cascades and reactive oxygen species bursts [12, 13, 14, 15, 16]. RLCKs have also been implicated in growth processes; for instance, mutations in BIK1 not only compromise immunity but also alter silique development as well as leaf and inflorescence morphology [17, 18, 19]. In rice, OsRLCK176, OsRLCK185, and OsRLCK107, the close homologs of AtBIK1 in rice, interact with CHITIN ELICITOR RECEPTOR KINASE 1 and are required for chitin and peptidoglycan signaling pathways [20, 21]. OsRLCK176 and OsRLCK118 participate in defense‐related cell death while modulating growth under the control of E3 ubiquitin ligase SPL11 and S‐domain RLK SPL11 cell‐death suppressor 2 (SDS2) [22]. OsRLCK185 interacts with and phosphorylates the C‐terminal regulatory domain of OsMAPKKKε to activate the mitogen‐activated protein kinase (MAPK) signaling cascade and positively regulate rice immunity [23, 24]. Recent studies further revealed that OsRLCK176 protein stability is dynamically regulated by OsCPK17‐mediated phosphorylation and OsPUB12‐dependent ubiquitination, enabling fine‐tuned immune homeostasis in response to pathogen‐associated molecular patterns [25].

MADS‐box transcription factors constitute a highly conserved family that governs floral organ specification, meristem identity, and reproductive development across angiosperms [26, 27]. In rice, OsMADS1 and OsMADS15 regulate spikelet determinacy, glume identity, and flower development, and their mutation leads to floral abnormalities and reduced fertility [28, 29, 30, 31, 32]. Beyond their classical roles, accumulating evidence indicates that MADS‐box genes also participate in environmental stress responses, including drought, cold, and pathogen challenge [33, 34, 35, 36, 37]. For instance, OsMADS26 negatively regulates immunity and drought tolerance in rice: its downregulation enhances resistance to *Magnaporthe oryzae* and *Xanthomonas oryzae*, whereas overexpression increases susceptibility [38]. In tobacco, silencing of *NbMADS1* compromises systemic resistance to *Phytophthora nicotianae* [39], while in wheat, the expression of *TaMADS19* and *TaMADS117* is dynamically altered during fungal infections [40]. These findings collectively highlight a broader regulatory role of MADS‐box transcription factors at the interface of development and immunity, yet how their stability and function are modulated during pathogen invasion remains largely unknown.

A growing body of evidence has implicated ubiquitin‐mediated protein degradation in coordinating defense and development. In *Arabidopsis*, E3 ubiquitin ligases such as PUB25/26 and PUB12/13 regulate the stability of immune kinases like BIK1 and receptors like FLS2 to fine‐tune PTI activation [41, 42]. Similarly, in rice, the S‐domain RLK SDS2 interacts with the E3 ubiquitin ligase SPL11 to regulate RLCKs and defense‐related programmed cell death, [22] while the E3 ubiquitin ligase IPI1 modulates both immunity and flowering by targeting distinct downstream substrates [43]. However, it remains unclear whether E3‐RLCK modules can simultaneously influence reproductive development and antiviral immunity through common signaling axes.

Our prior work revealed that P3IP1, a virus‐inducible RING‐type E3 ubiquitin ligase, is transcriptionally activated by the RGSV‐encoded P3 protein and subsequently phosphorylated by SERK4, resulting in the degradation of NRPD1a and suppression of RNA‐directed DNA methylation (RdDM)‐mediated antiviral defense [44, 45]. In transgenic rice, overexpression of P3IP1 induces dwarfism, tillering, and fertility defects similar to RGSV symptoms. While the immune‐related effects of P3IP1 have been partially elucidated, the mechanism underlying its reproductive abnormalities, particularly the reduction in pollen viability and seed setting, remains unclear [45]. In this study, we reveal that the virus‐inducible E3 ubiquitin ligase P3IP1 facilitates the ubiquitin–proteasome–mediated degradation of the receptor‐like cytoplasmic kinase RLCK22. RLCK22 directly interacts with and stabilizes the floral MADS‐box transcription factors MADS1 and MADS15, which are required for pollen viability and contribute to antiviral defense. Loss of *RLCK22* leads to the destabilization of MADS1/15, resulting in impaired male fertility and increased viral susceptibility. Through a combination of genetic, biochemical, and in vivo interaction assays, we demonstrate that P3IP1, RLCK22, and MADS1/15 are associated in vivo. Moreover, P3‐driven upregulation of P3IP1 promotes the degradation of RLCK22 and MADS1/15. Together, our findings uncover a hierarchical protein degradation cascade that connects viral effector sensing with the coordinated repression of reproductive development and innate immunity via post‐translational regulation.

## 2 Results

### 2.1 Overexpression of P3IP1 Reduces Seed Setting and Pollen Viability via Destabilization of MADS1 and MADS15 Through an Indirect Pathway

**FIGURE 1:**
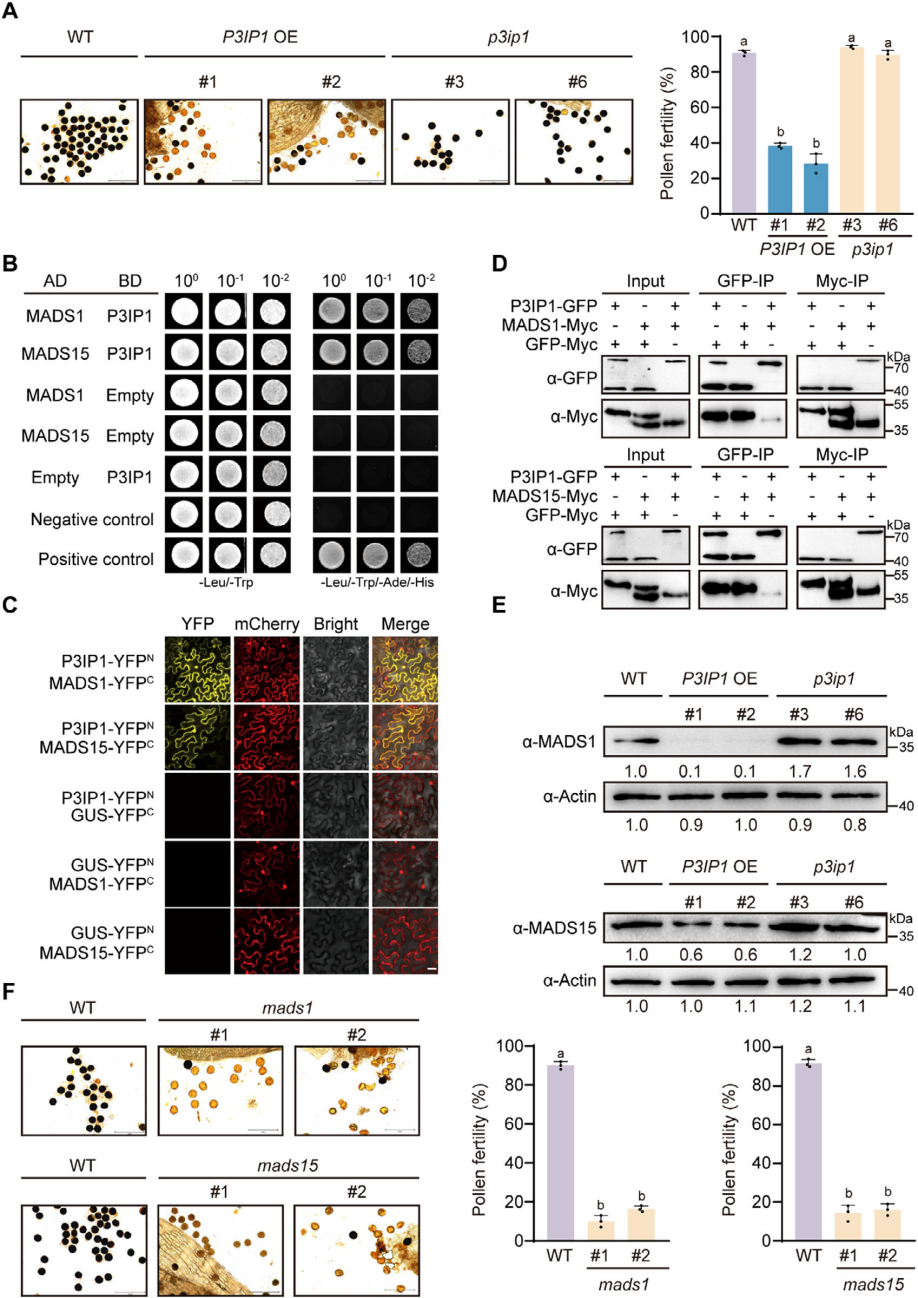
Identification of interactions between P3IP1 and MADS1/15 and their effects on pollen viability. (A) I_2_‐KI staining and pollen fertility statistical analysis of WT, *P3IP1* OE, and *p3ip1* rice plants. Pollen grains staining black were scored as viable, and those staining yellow or light red were considered sterile (*n* = 3). Bar = 150 µm. (B) Yeast two‐hybrid assays were conducted to detect protein–protein interactions between P3IP1 and MADS1 or MADS15. (C) BiFC assays were performed in *N. benthamiana* to confirm the interactions between P3IP1 and MADS1 or MADS15. mCherry was used as a nuclear localization marker. Bar = 50 µm. (D) Co‐IP assays were used to validate the interactions between P3IP1 and MADS1/15 in *N. benthamiana*. (E) Immunoblot analysis of MADS1 and MADS15 protein levels in WT, *P3IP1* OE, and *p3ip1* rice plants. Actin was used as an internal control. (F) I_2_‐KI staining and pollen fertility statistical analysis of WT, *mads1*, and *mads15* rice plants. Pollen grains staining black were judged as viable, and those staining yellow or light red were considered sterile (*n* = 3). Bar = 150 µm. Data were presented as mean ± *SD*. Different lowercase letters above bars indicate significant differences (*p* < 0.05) based on a one‐way ANOVA test.

To investigate the biological function of the E3 ubiquitin ligase P3IP1 in rice development, we first examined the phenotypes of transgenic lines overexpressing *P3IP1* (*P3IP1* OE) and CRISPR/Cas9‐mediated knockout mutants (*p3ip1*) (Figure S1A,B). Compared with wild‐type (WT) plants, *P3IP1* OE lines exhibited significantly reduced seed setting rate, shorter panicles, decreased floret number and primary panicle branch length, whereas *p3ip1* plants showed no obvious defects (Figure S1C–I). These observations suggested a possible role of P3IP1 in reproductive development. To explore the underlying cause of reduced fertility, we examined pollen viability by potassium iodide (KI) staining. The proportion of viable pollen was markedly lower in *P3IP1* OE plants compared to WT and *p3ip1* lines (Figure 1A), indicating that *P3IP1* overexpression impairs pollen viability. To gain mechanistic insight, we sought to identify potential P3IP1‐interacting proteins involved in reproductive development. Yeast two‐hybrid (Y2H) screening revealed that P3IP1 interacts with two key MADS‐box transcription factors, MADS1 and MADS15 (Figure 1B), which are essential regulators of floral organ identity and pollen development. [28, 29, 32] These interactions were further validated by bimolecular fluorescence complementation (BiFC) assays (Figure 1C; Figure S2A,B) and co‐immunoprecipitation (Co‐IP) assay in *Nicotiana benthamiana* (*N. benthamiana*) (Figure 1D). We next assessed whether P3IP1 affects the protein stability of MADS1 and MADS15. Western blot analysis showed that both proteins accumulated at markedly lower levels in *P3IP1* OE plants, while their levels were significantly elevated in *p3ip1* lines (Figure 1E), suggesting that P3IP1 is associated with reduced MADS1/15 protein abundance.

To test whether P3IP1 directly affects MADS1/15 protein abundance, we performed transient co‐expression assays in *N. benthamiana*. Increasing the dosage of P3IP1‐HA did not alter the abundance of Myc‐tagged MADS1 or MADS15 (Figure S2C,E). In vitro ubiquitination assays further confirmed that P3IP1 cannot directly ubiquitinate either MADS1 or MADS15 (Figure S2D,F), implying that an intermediate factor may mediate this effect. Functionally, CRISPR knockout lines of MADS1 (*mads1*) and MADS15 (*mads15*) also exhibited significantly reduced pollen viability (Figure 1F), consistent with their established roles in male fertility and further supporting that P3IP1 may exert its influence on reproduction through destabilization of these transcription factors. Together, these findings show that overexpression of P3IP1 is associated with impaired fertility and reduced pollen viability in rice, accompanied by decreased MADS1 and MADS15 protein levels.

### 2.2 RLCK22 Interacts With and Stabilizes MADS1/15, Associating With Pollen Viability

**FIGURE 2:**
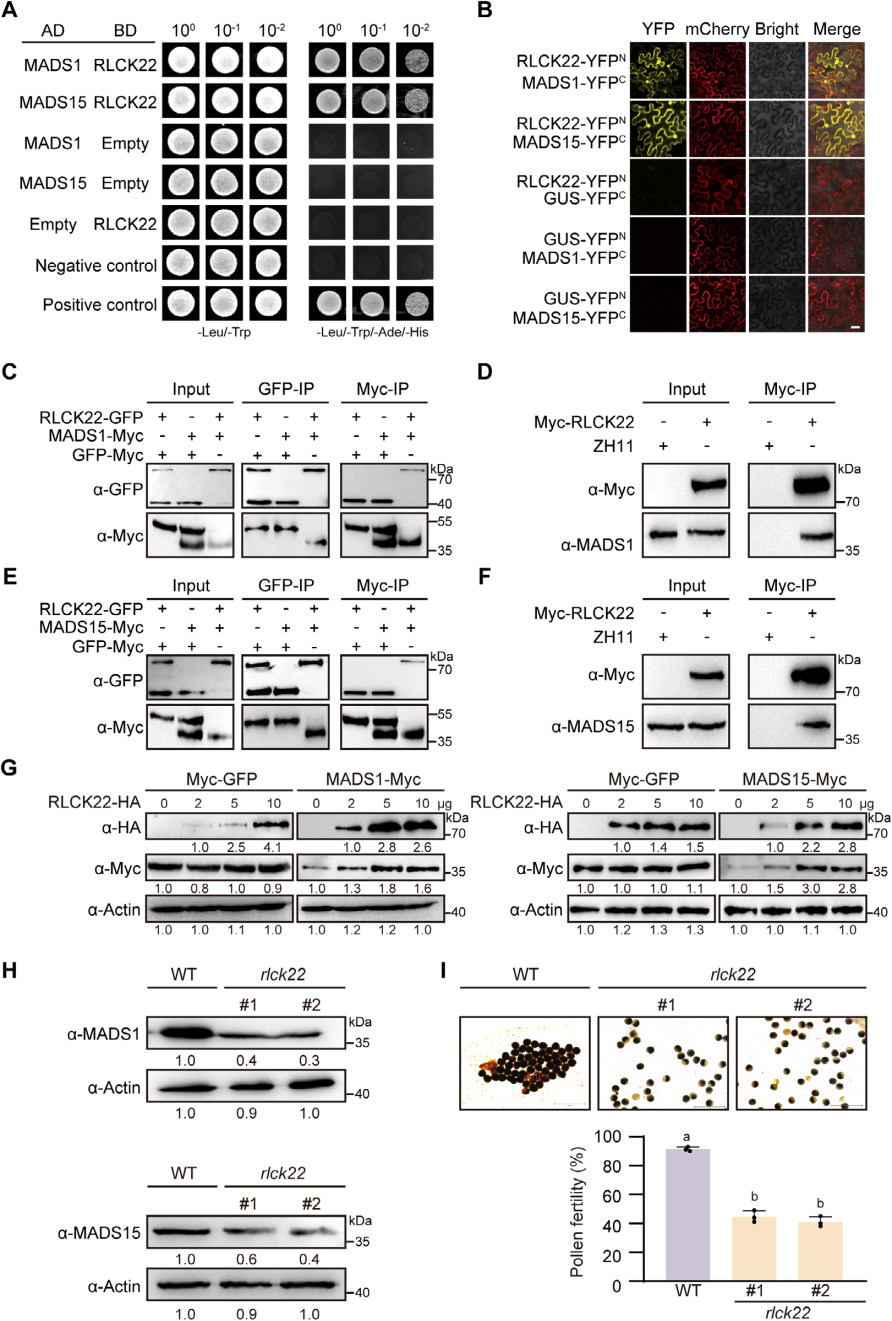
Interactions between RLCK22 and MADS1/15. (A) Yeast two‐hybrid assays were conducted to detect protein–protein interactions between RLCK22 and MADS1 or MADS15. (B) BiFC assays were performed in *N. benthamiana* to confirm the interactions between RLCK22 and MADS1 or MADS15. mCherry was used as a plasma membrane localization marker. Bar = 50 µm. (C,E) Co‐IP assays were used to validate the interactions between RLCK22 and MADS1 C) or MADS15 E) in *N. benthamiana*. (D,F) In vivo pull‐down assays using Myc‐RLCK22 transgenic rice plants to assess protein interaction. (G) Transient expression assays in *N. benthamiana* showing dose‐dependent increased accumulation of MADS1‐Myc (left) and MADS15‐Myc (right) levels upon co‐expression with increasing amounts of RLCK22‐HA. Myc‐GFP was used as a control. (H) Immunoblot analysis of MADS1 and MADS15 protein levels in WT and *rlck22* rice plants. Actin was used as an internal control. (I) I_2_‐KI staining and pollen fertility statistical analysis of WT and *rlck22* rice plants. Pollen grains staining black were judged as viable, and those staining yellow or light red were considered sterile (*n* = 3). Bar = 150 µm. Data were presented as mean ± *SD*. Different lowercase letters above bars indicate significant differences (*p* < 0.05) based on a one‐way ANOVA test.

To elucidate how P3IP1 affects the stability of MADS1 and MADS15, we next investigated potential regulatory proteins that interact with these transcription factors. Yeast two‐hybrid assays showed that a receptor‐like cytoplasmic kinase, RLCK22, physically interacts with both MADS1 and MADS15 (Figure 2A). These interactions were further confirmed in planta using BiFC assays (Figure 2B), Co‐IP experiments (Figure 2C,E), and in vivo pull‐down assays (Figure 2D,F), all of which supported an association between RLCK22 and MADS1/15.

Given that RLCK22 is predicted to be a kinase, we examined whether it could phosphorylate its interacting partners. In vitro kinase assays confirmed that RLCK22 possesses auto‐phosphorylation activity (Figure S3). However, no phosphorylation signals were detected for either MADS1 or MADS15 in the presence of RLCK22 (Figure S3A,B), suggesting that this association does not involve detectable phosphorylation of MADS1 or MADS15. To explore the functional relationship between RLCK22 and MADS1/15, we performed transient co‐expression assays in *N. benthamiana*. Increasing RLCK22‐HA dosage led to a dose‐dependent accumulation of MADS1‐Myc and MADS15‐Myc proteins, while Myc‐GFP control remained unchanged (Figure 2G), suggesting that RLCK22 is associated with increased MADS1 and MADS15 protein abundance.

We next generated CRISPR/Cas9 knockout lines of *RLCK22* and obtained two independent homozygous mutants (*rlck22* #1 and #2) with confirmed deletions (Figure S4A). The *rlck22* mutants displayed no obvious differences from WT plants in terms of overall morphology, except for a significant increase in grain thickness compared with WT (Figure S4B–D). However, immunoblotting revealed that endogenous MADS1 and MADS15 protein levels were markedly reduced in *rlck22* mutants compared to WT plants (Figure 2H). Furthermore, potassium iodide staining revealed that the *rlck22* mutants exhibited significantly reduced pollen viability compared to WT plants (Figure 2I). These results indicate that RLCK22 is required for normal pollen viability and is associated with reduced MADS1 and MADS15 protein levels when disrupted.

### 2.3 P3IP1 Targets RLCK22 for Ubiquitin‐Mediated Degradation

**FIGURE 3:**
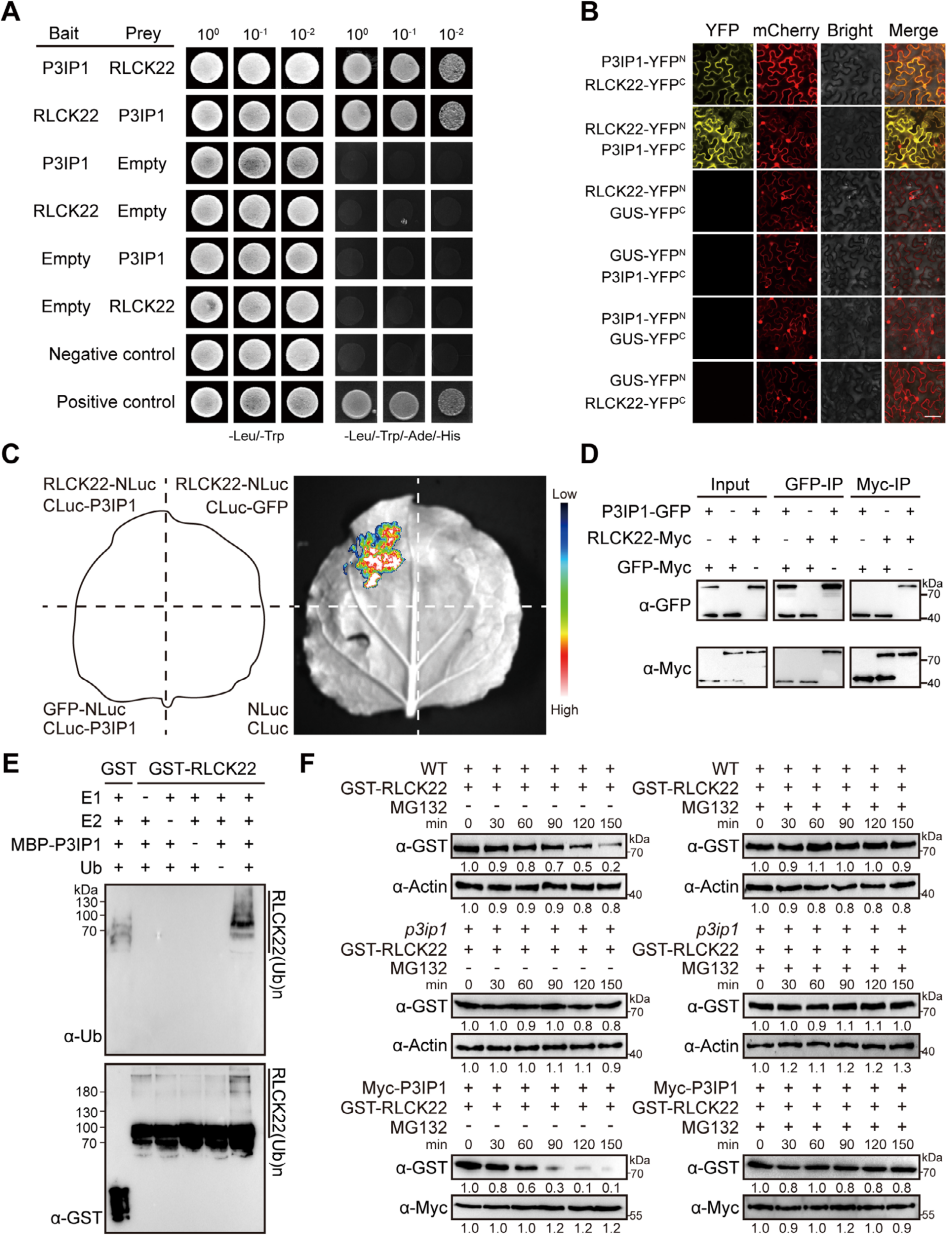
Identification of RLCK22 as a direct substrate of P3IP1. (A) Yeast two‐hybrid assay showing the interaction between P3IP1 and RLCK22. (B) BiFC assay in *N. benthamiana* leaves to verify the interaction between P3IP1 and RLCK22. Bar = 50 µm. (C) Split‐luciferase complementation assay to confirm the interaction between P3IP1 and RLCK22 in *N. benthamiana*. (D) Co‐IP assay detecting the interaction between P3IP1 and RLCK22 in *N. benthamiana*. (E) In vitro ubiquitination assay examining ubiquitination of RLCK22 in the presence of P3IP1. (F) In vitro degradation assay using total protein extracts from WT, Myc‐P3IP1 overexpression plants and *p3ip1* mutant rice and purified GST‐RLCK22 at various time points (0–150 min) without (left) or with (right) MG132 treatment.

Having identified RLCK22 as a physical interactor of MADS1/15, we next examined whether RLCK22 is regulated by P3IP1. Yeast two‐hybrid assays identified RLCK22 as a potential interactor of P3IP1 (Figure 3A), and the interaction between P3IP1 and RLCK22 was further validated by BiFC (Figure 3B), split‐luciferase complementation (Figure 3C), and Co‐IP assays (Figure 3D). In vitro ubiquitination assays demonstrated that P3IP1 can promote ubiquitination of RLCK22 (Figure 3E). To determine whether P3IP1 mediates RLCK22 degradation, total protein extracts from WT, *p3ip1* mutant, and P3IP1‐overexpressing rice plants (Myc‐P3IP1) were incubated with purified GST‐RLCK22 protein. Western blot analysis showed a time‐dependent decrease in RLCK22 protein levels during incubation. RLCK22 degradation was most pronounced in extracts from *P3IP1* OE plants, occurred to a lesser extent in WT extracts, and was largely abolished in extracts from *p3ip1* mutants, supporting a role for P3IP1 in RLCK22 degradation. In all cases, RLCK22 degradation was suppressed by the proteasome inhibitor MG132 (Figure 3F), suggesting that P3IP1 mediates RLCK22 degradation via the ubiquitin–proteasome pathway. Since RLCK22 is a putative receptor‐like cytoplasmic kinase, we examined whether it could phosphorylate P3IP1. In vitro kinase assays using a phospho‐specific antibody showed that no detectable phosphorylation signal was observed for P3IP1 (Figure S3C), indicating that RLCK22 does not phosphorylate P3IP1 under the conditions tested.

### 2.4 P3IP1 Indirectly Promotes the Degradation of MADS1 and MADS15 Through RLCK22

**FIGURE 4:**
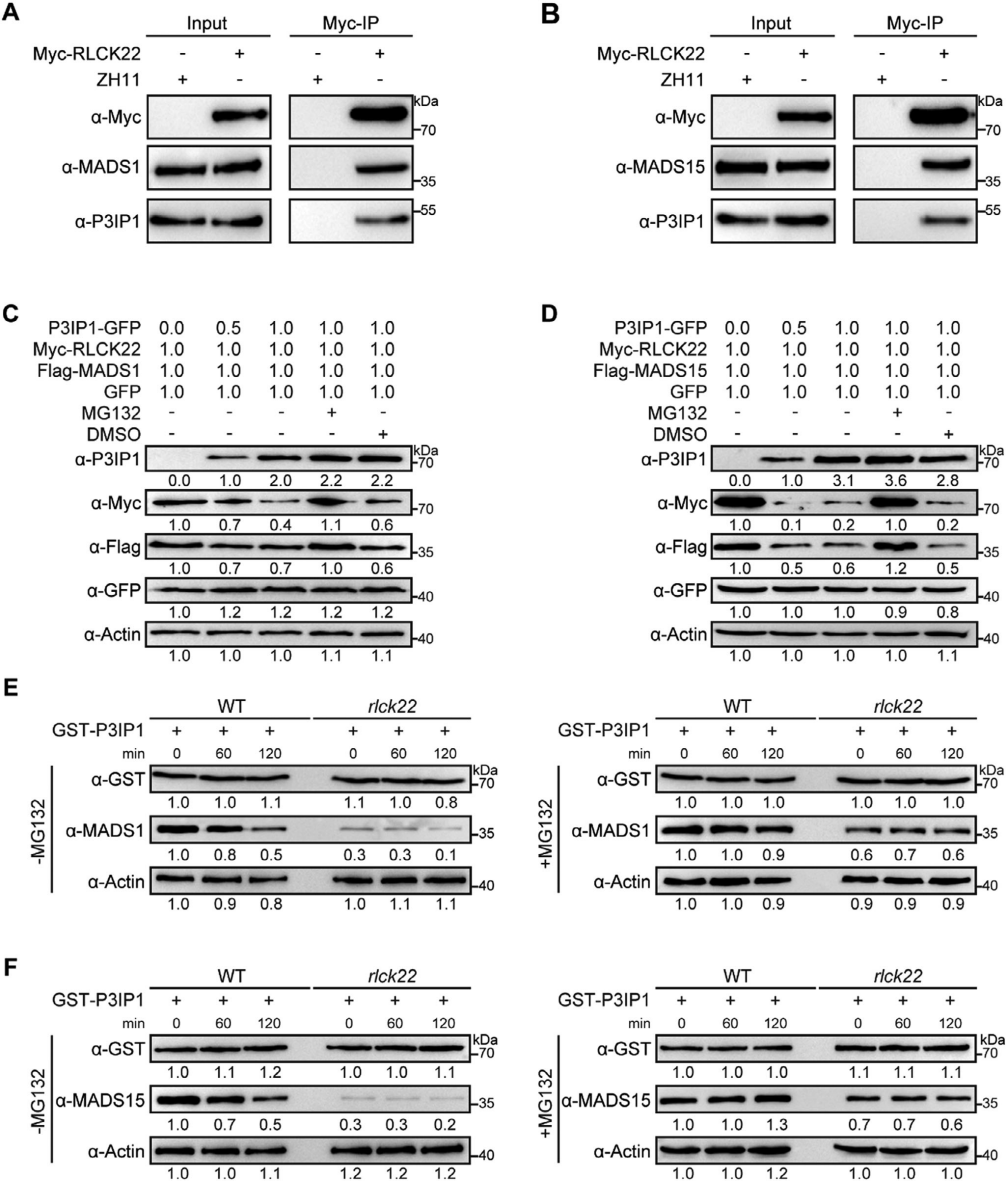
P3IP1 is associated with RLCK22‐dependent regulation of MADS1 and MADS15 stability. (A,B) In vivo Myc pull‐down assay using Myc‐RLCK22 transgenic rice plants to assess protein interaction. (C,D) Co‐expression of P3IP1‐GFP, Myc‐RLCK22, and Flag‐MADS1 C) or Flag‐MADS15 D) in *N. benthamiana* to assess RLCK22 and MADS1/15 protein levels with increasing amounts of P3IP1‐GFP with or without MG132 treatment. Actin was used as an internal control. (E,F) Degradation assays showing time‐dependent protein levels of endogenous MADS1 E) or MADS15 F) upon incubation with purified GST‐P3IP1 and total protein extracts from WT or *rlck22* rice without (left) or with (right) MG132 treatment. Actin was used as internal control.

To determine whether P3IP1, RLCK22, and MADS1/15 assemble into a protein complex, we conducted in vivo Myc pull‐down assays using transgenic rice plants expressing Myc‐tagged RLCK22. Both endogenous P3IP1 and MADS1/15 proteins were detected in the Myc–RLCK22 immunoprecipitates, indicating that RLCK22 is associated with P3IP1 and MADS1/15 in rice cells (Figure 4A,B). To determine whether P3IP1 regulates MADS1 through RLCK22, we conducted transient co‐expression assays in *N. benthamiana*. Increasing expression of P3IP1‐GFP led to a dose‐dependent reduction in the protein levels of both RLCK22 and MADS1/15. Notably, this reduction was reversed upon treatment with the proteasome inhibitor MG132 (Figure 4C,D), suggesting that the degradation process is proteasome‐dependent. To further confirm that P3IP1‐induced degradation of MADS1/15 depends on RLCK22, we designed a cell‐free degradation assay. Total protein extracts were prepared from WT and *rlck22* mutant rice plants and incubated with purified GST‐P3IP1 for 0, 60, or 120 min. At each time point, the levels of endogenous MADS1 and MADS15 proteins were detected by immunoblotting using Actin as a loading control. In WT extracts, the levels of endogenous MADS1 and MADS15 gradually decreased over time, whereas no change was observed in *rlck22* mutant extracts (Figure 4E,F). Moreover, the addition of MG132 abolished the time‐dependent reduction of MADS1/15 in WT extracts, but had no effect in *rlck22* mutants (Figure 4E,F). These results confirm that P3IP1 promotes the degradation of MADS1 and MADS15 through an RLCK22‐dependent, proteasome‐mediated mechanism. Together, these findings establish that P3IP1 indirectly destabilizes MADS1 and MADS15 by targeting RLCK22 for degradation, revealing a hierarchical protein turnover pathway that links virus‐induced E3 ubiquitin ligase activity to impaired reproductive development.

### 2.5 RGSV P3 Promotes MADS1/15 Degradation Through P3IP1‐RLCK22, Enhancing Susceptibility

**FIGURE 5:**
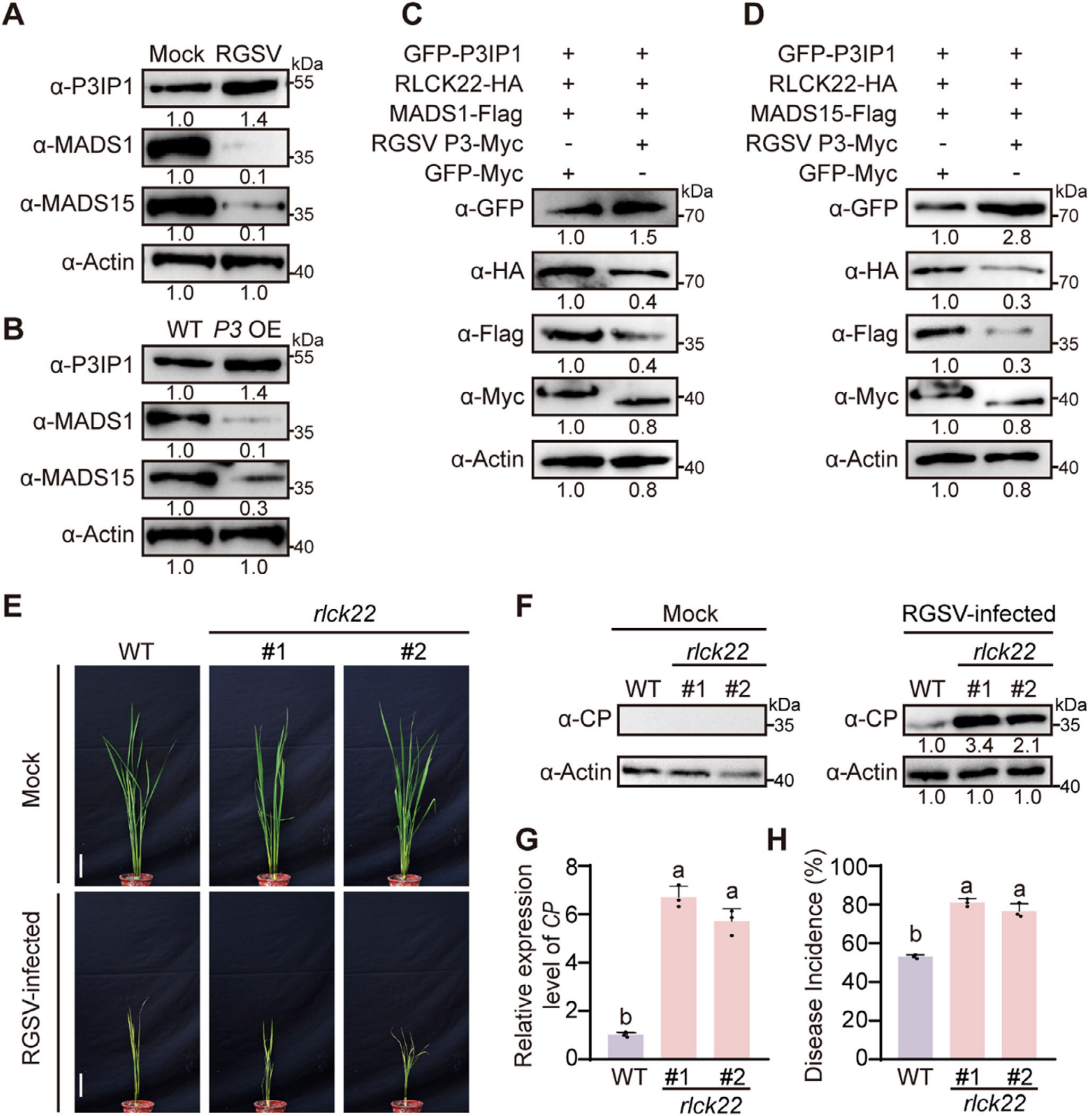
RGSV P3 is associated with reduced RLCK22–MADS1/15 protein levels and altered antiviral responses in rice. (A) Immunoblot analysis showing protein levels of MADS1 and MADS15 in mock‐ or RGSV‐infected rice at 4 weeks post‐inoculation (wpi). Actin was used as an internal control. (B) Immunoblot detection of MADS1 and MADS15 in WT and *P3* OE rice lines. Actin was used as an internal control. (C) and D) Transient co‐expression of GFP‐P3IP1, RLCK22‐HA, and MADS1‐Flag C) or MADS15‐Flag D) with P3‐Myc or control GFP‐Myc in *N. benthamiana*. Actin was used as an internal control. (E) Phenotypic comparison of WT and two independent *rlck22* mutant lines (#1, #2) upon mock‐inoculation or RGSV infection at 4 wpi. Bar = 10 cm. (F) Immunoblot analysis of RGSV CP in WT and *rlck22* mutant plants at 4 wpi. Actin was used as an internal control. (G) RT‐qPCR quantification of *CP* transcripts in WT and *rlck22* mutants at 4 wpi. Expression normalized to *OsEF1α* (*n* = 3). (H) Disease incidence of WT and *rlck22* mutant lines infected with RGSV (*n* = 3). Data were presented as mean ± *SD*. Different lowercase letters above bars indicate significant differences (*p* < 0.05) based on a one‐way ANOVA test.

Our previous study demonstrated that the RGSV‐encoded P3 protein activates the expression of P3IP1, which functions as an E3 ubiquitin ligase [44, 45]. Given our current findings that P3IP1 mediates the degradation of RLCK22, thereby reducing the stability of MADS1 and MADS15, we next investigated whether RGSV infection or P3 overexpression could modulate MADS1 and MADS15 protein levels. In RGSV‐infected rice, immunoblotting revealed a strong induction of P3IP1 expression, accompanied by a marked decrease in both MADS1 and MADS15 protein levels compared to mock‐inoculated plants (Figure 5A). Similarly, in P3‐overexpressing transgenic rice, the accumulation of P3IP1 protein was significantly increased, while the levels of MADS1 and MADS15 were substantially reduced relative to WT plants (Figure 5B). To further examine whether P3 acts upstream of the P3IP1‐RLCK22‐MADS module, we performed transient expression assays in *N. benthamiana*. Co‐expression of P3IP1‐GFP, RLCK22‐HA, and MADS1‐Flag with P3‐Myc resulted in higher levels of P3IP1‐GFP and lower levels of RLCK22‐HA and MADS1‐Flag, compared to control samples expressing GFP‐Myc (Figure 5C). A similar reduction in RLCK22‐HA and MADS15‐Flag was observed when co‐expressed with P3‐Myc in the presence of P3IP1‐GFP (Figure 5D), supporting that P3 promotes the reduction of RLCK22 and MADS1/15 protein levels in a P3IP1‐associated manner. To evaluate the biological relevance of this pathway in antiviral immunity, we inoculated two independent *rlck22* mutant lines (#1 and #2) and *RLCK22* OE rice lines with RGSV and assessed disease phenotypes four weeks post‐inoculation (wpi). Compared with WT plants, the *rlck22* mutants exhibited enhanced dwarfing symptoms, whereas *RLCK22* OE plants showed markedly attenuated disease symptoms following RGSV infection (Figure 5E; Figure S4E,F). Consistently, immunoblot analysis revealed that viral coat protein (CP) accumulation was significantly increased in *rlck22* mutants but reduced in *RLCK22* OE plants relative to WT (Figure 5F; Figure S4G). These trends were further supported by RT–qPCR analysis of RGSV CP transcript levels (Figure 5G; Figure S4H). Moreover, statistical analysis of disease incidence at 6 wpi showed that *rlck22* mutants displayed a significantly higher disease incidence, whereas RLCK22 OE plants exhibited a lower disease incidence compared with WT controls (Figure 5H; Figure S4I). Collectively, these results demonstrate that the RGSV‐encoded P3 protein activates the P3IP1‐RLCK22‐MADS1/MADS15 degradation cascade, and that RLCK22 plays a key role in restricting RGSV infection.

### 2.6 MADS1 and MADS15 Positively Regulate Antiviral Immunity Against RGSV via Transcriptional Reprogramming

**FIGURE 6:**
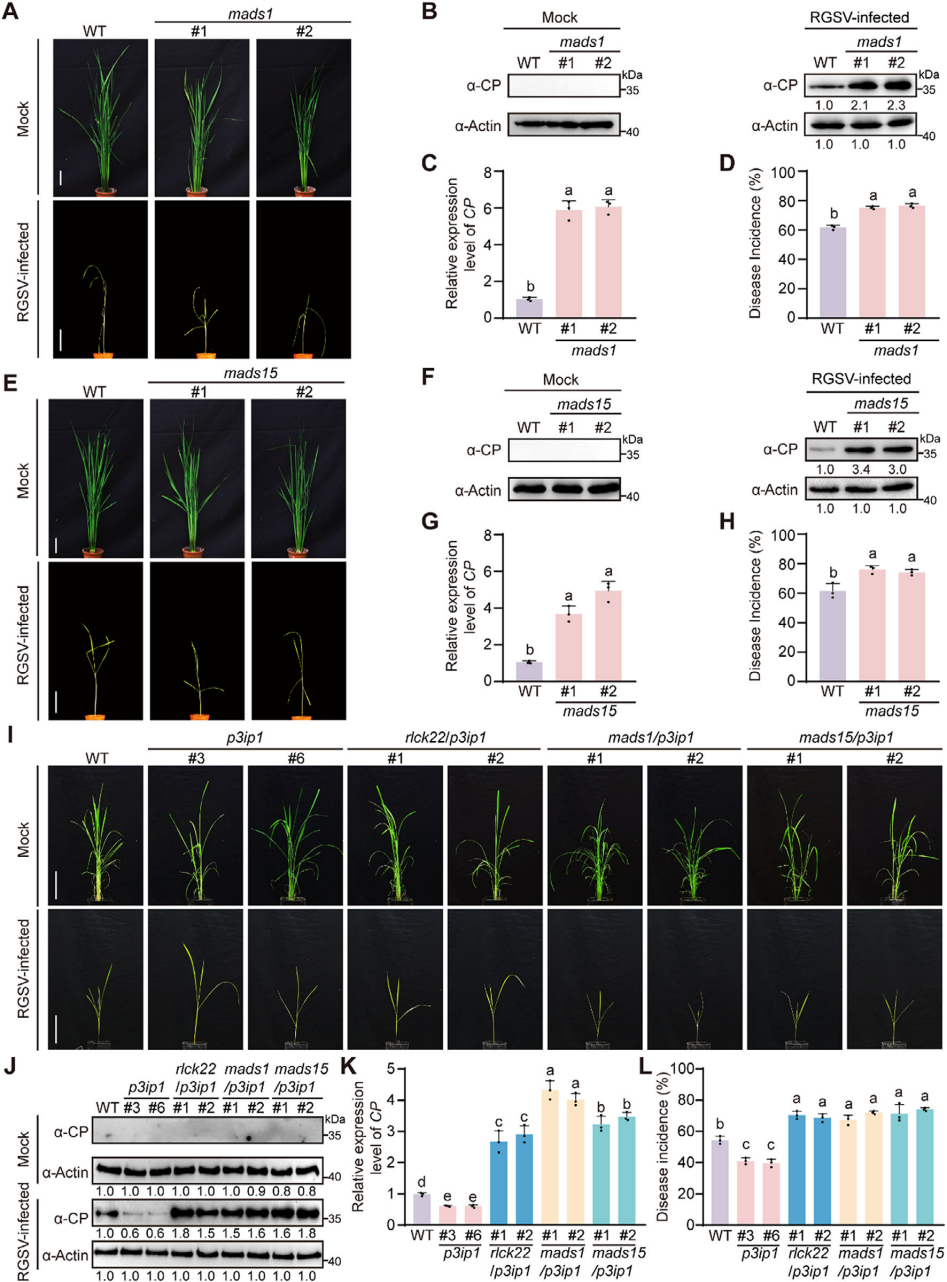
RGSV susceptibility in *mads1*, *mads15*, and related mutant combinations. (A,E) Phenotypic comparison of WT and two independent *mads1* mutant lines (#1, #2) A) or *mads15* mutant lines (#1, #2) E) upon mock‐inoculation or RGSV infection at 4 wpi. Bar = 10 cm. (B,F) Immunoblot analysis of RGSV CP in WT and *mads1* mutant B) or *mads15 mutant* F) plants at 4 wpi. Actin was used as an internal control. (C) and G) RT‐qPCR quantification of *CP* transcripts in WT and *mads1* mutant C) or *mads15 mutant* G) at 4 wpi. Expression normalized to *OsEF1α* (*n* = 3). (D,H) Disease incidence of WT and *mads1* mutant D) or *mads15 mutant* H) plants infected with RGSV (*n* = 3). (I) Phenotypic comparison of WT, *p3ip1* and double mutant rice plants upon mock‐inoculation or RGSV infection at 4 wpi. Bar = 10 cm. (J) Immunoblot analysis of RGSV CP in WT and indicated plants at 4 wpi. Actin was used as an internal control. (K) RT‐qPCR quantification of *CP* transcripts in WT and indicated plants at 4 wpi. Expression normalized to *OsEF1α* (*n* = 3). (L) Disease incidence of WT and indicated plants infected with RGSV (*n* = 3). Data were presented as mean ± *SD*. Different lowercase letters above bars indicate significant differences (*p* < 0.05) based on a one‐way ANOVA test.

To assess the involvement of MADS1 and MADS15 in antiviral defense against RGSV, CRISPR/Cas9 knockout lines and OE lines for each gene were generated (Figure S5A). Upon RGSV inoculation, *mads1* and *mads15* mutants exhibited more severe dwarfing symptoms than WT plants, whereas *MADS1* and *MADS15* OE plants showed attenuated disease symptoms at 4 wpi (Figure 6A,E; Figure S5B,C,G,H). Consistently, immunoblot analyses revealed increased accumulation of viral CP in *mads* mutants but reduced CP levels in OE plants relative to WT (Figure 6B,F; Figure S5D,I), which was further supported by RT–qPCR analyses of CP transcript levels (Figure 6C,G; Figure S5E,J). Moreover, disease incidence surveys at 6 wpi showed higher disease incidence in *mads* mutants and lower incidence in OE plants compared with WT controls (Figure 6D,H; Figure S5F,K), supporting a positive role for MADS1 and MADS15 in RGSV resistance. To further examine the genetic relationships among P3IP1, RLCK22, and MADS1/15, we generated a series of double mutants, including *rlck22*/*p3ip1*, *mads1*/*p3ip1*, *mads15*/*p3ip1*, *mads1*/*rlck22*, and *mads15*/*rlck22*. Following RGSV inoculation, all double mutants displayed compromised resistance to viral infection, exhibiting disease symptoms, viral CP accumulation, and disease incidence comparable to those observed in *rlck22* and *mads* single mutants (Figure 6I–L; Figure S6). Notably, the enhanced resistance phenotype of *p3ip1* was not observed in any of the double mutant backgrounds, indicating that RLCK22 and MADS1/15 are required for the antiviral resistance conferred by *p3ip1*.

To further investigate the molecular basis of the antiviral functions of MADS1 and MADS15, transcriptome profiling was performed in *mads1* and *mads15* mutants before and after RGSV infection. RNA‐seq analyses revealed that RGSV‐induced transcriptional reprogramming was markedly altered in both mutants, with a predominant reduction in the induction of genes associated with defense‐related responses and metabolic processes (Figure S7, Tables S1 and S2). Notably, *mads1* and *mads15* mutants exhibited highly overlapping transcriptional changes, indicating that these two MADS‐box factors regulate similar downstream pathways during antiviral responses. These transcriptomic data collectively suggest that both MADS1 and MADS15 contribute to antiviral defense by regulating genes involved in defense response, detoxification, and metabolic remodeling. To validate the RNA‐seq findings, we selected 20 representatives differentially expressed genes (DEGs) for RT‐qPCR analysis. Most of these genes showed no significant changes in WT plants upon infection but were markedly altered in *mads1* and *mads15* mutants (Figure S8), indicating that these genes may be downstream targets of MADS1 and MADS15 and participate in the regulation of antiviral immunity.

## 3 Discussion

**FIGURE 7:**
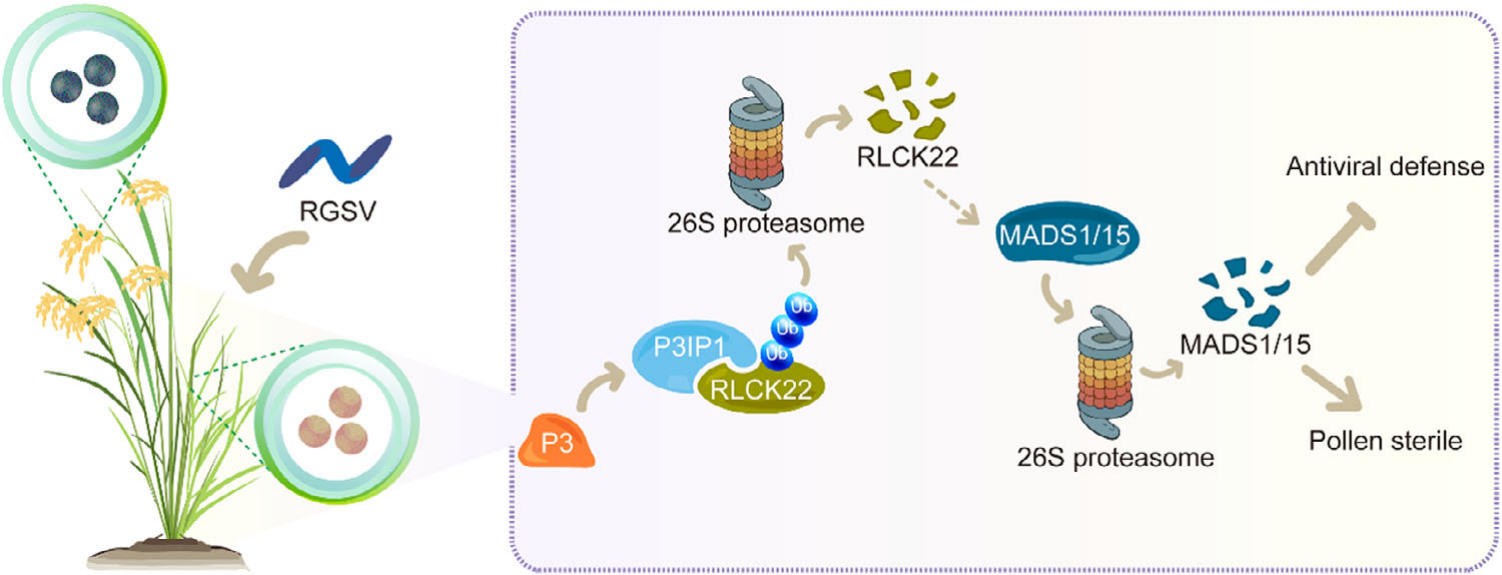
A proposed model illustrating the hierarchical degradation cascade mediated by P3IP1 in rice. Upon infection by RGSV, the viral P3 protein transcriptionally activates the host RING‐type E3 ubiquitin ligase P3IP1. Induced P3IP1 interacts with and ubiquitinates the receptor‐like cytoplasmic kinase RLCK22, leading to its degradation via the 26S proteasome pathway. RLCK22 normally interacts with and stabilizes the floral MADS‐box transcription factors MADS1 and MADS15. Loss of RLCK22 results in the destabilization of MADS1/15, thereby impairing both pollen viability and antiviral immunity. This cascade represents a viral strategy to coordinately suppress host reproductive development and immune responses by hijacking a core post‐translational regulatory module.

Viruses frequently manipulate host cellular mechanisms to facilitate infection, yet the molecular interplay linking viral effectors to suppression of both plant immunity and reproductive development remains poorly understood. In this study, we reveal that RGSV infection induces the E3 ubiquitin ligase P3IP1, which targets RLCK22 for ubiquitin‐dependent proteasomal degradation, leading to the destabilization of two MADS‐box transcription factors essential for pollen viability and antiviral defense (Figure 7). This hierarchical degradation cascade identifies a distinct viral strategy co‐opting developmental regulators to coordinate dual suppression of immunity and fertility.

P3IP1 has been previously implicated in RdDM suppression via ubiquitination of NRPD1a [44, 45]. Interestingly, our findings suggest that P3IP1 may also exert broader regulatory roles. We now show it also targets RLCK22‐a receptor‐like cytoplasmic kinase for degradation (Figure 3). Despite retaining auto‐kinase activity, RLCK22 does not phosphorylate P3IP1 or MADS proteins (Figure S3), suggesting a non‐catalytic, scaffold‐like function that contributes to stabilize a multi‐protein complex. Such non‐enzymatic scaffolding roles have been described for various kinases in both animal and plant signaling networks [46, 47, 48] yet remain largely underexplored among plant RLCKs, particularly in reproductive contexts. RLCKs, especially those in RLCK‐VII subfamily, have emerged as central signaling hubs downstream of pattern recognition receptors (PRRs) and RLKs, integrating developmental and immune pathways [9, 16, 49, 50, 51, 52, 53]. Our study adds a new dimension to their functionality by linking RLCK22 to transcriptional regulation of developmental and defense responses via MADS‐box proteins. The destabilization of MADS1 and MADS15 upon RLCK22 loss highlights a unique layer of transcriptional control vulnerable to pathogen interference.

Beyond their established roles in floral organ identity and spikelet determinacy [28, 54, 55, 56, 57], MADS1 and MADS15 are shown here to participate in antiviral defense (Figure 6). Knockout mutants of these genes displayed increased RGSV susceptibility, accompanied by widespread transcriptomic remodeling in genes related to biotic response, secondary metabolism, and detoxification pathways—a hallmark of viral infection–induced metabolic reprogramming (Figures S7 and S8). Similar functions of developmental transcription factors in pathogen responses have been documented in different plants [38, 39, 40, 58, 59, 60, 61], underscoring a broader regulatory interface between development and immunity. Our data are consistent with broader themes in plant–pathogen interactions, where viruses subvert hormonal pathways, reactive oxygen homeostasis, and nutrient allocation to promote infection [62]. For example, phytoplasmas and certain viruses reprogram floral organogenesis to induce sterility, often via targeting floral transcription factors or hormone pathways [63, 64]. The RGSV‐triggered degradation of MADS1/15 may reflect a similar virulence strategy that compromises both reproduction and defense by hijacking host developmental circuits.

Despite establishing a hierarchical degradation pathway linking P3IP1, RLCK22, and MADS1/15, a critical unresolved question is how RLCK22 contributes to the stabilization of these MADS‐box transcription factors. Given the absence of direct phosphorylation, RLCK22 may function as a non‐catalytic scaffold that facilitates complex stability or protects its partners from proteasome‐mediated degradation [8, 49, 65]. Such non‐enzymatic roles for kinases have been increasingly recognized in plants and other systems, where kinases act as organizational hubs rather than active enzymes [47, 66, 67, 68]. In this context, additional co‐regulatory components—such as chromatin‐associated remodelers, protein quality control factors, or nuclear trafficking regulators—may be involved in modulating the accessibility or turnover of MADS1/15 proteins. Identifying these intermediate players will be essential to fully elucidate how viral manipulation of RLCK22 leads to developmental and immune disruption.

## 4 Conclusion

In summary, this study reveals a sophisticated viral strategy that co‐opts a developmental‐immunity signaling scaffold to coordinately dismantle plant reproductive and immune systems. By activating the host E3 ubiquitin ligase P3IP1, the Rice grassy stunt virus effector P3 initiates a hierarchical degradation cascade that destabilizes RLCK22 and its associated MADS‐box transcription factors MADS1 and MADS15—key regulators of pollen viability and antiviral defense. This post‐translational regulatory axis illustrates how pathogens exploit the convergence point of developmental and immune pathways to maximize infection success. Beyond advancing our mechanistic understanding of host–pathogen interplay, these findings identify actionable molecular targets for the development of virus‐resilient crops, offering new opportunities to enhance plant resistance without compromising reproductive capacity.

## 5 Experimental Section

### 5.1 Plant Growth Conditions

Seeds of wild‐type (WT) *Oryza sativa* ssp. *japonica* cv. Zhonghua11 (ZH11) and transgenic lines were germinated and grown in a controlled growth room maintained at 28°C with 60 ± 5% relative humidity under a 14/10 h (day/night) photoperiod prior to phenotypic and molecular analyses. *N. benthamiana* plants were grown in a growth chamber under a 16/8 h (day/night) photoperiod at 25°C for transient gene expression assays.

### 5.2 Plasmids and Genetic Transformation

The coding sequences of *RLCK22* (LOC_Os01g04520), *MADS1* (LOC_Os03g11614), and *MADS15* (LOC_Os07g01820) were amplified by reverse transcription PCR (RT‐PCR). CRISPR/Cas9 constructs targeting *RLCK22*, *MADS1*, and *MADS15* were generated using the pYLCRISPR/Cas9 vector backbone [69]. The constructs were introduced into ZH11 rice plants via *Agrobacterium tumefaciens*‐mediated transformation (BioRun, Wuhan, China). Homozygous CRISPR/Cas9 knockout lines were validated by bidirectional Sanger sequencing and analyzed using SnapGene software (https://www.snapgene.com). Primer sequences are provided in Table S3.

### 5.3 Virus Inoculation

Virus inoculation was performed as previously described, with minor modifications [45]. Briefly, rice seedlings were cultivated in a greenhouse at 28°C–30°C with 60% ±  5% relative humidity for 10 days. Viruliferous and non‐viruliferous brown planthoppers (*Nilaparvata lugens*, BPH) were then transferred onto the seedlings and allowed to feed for 3 days in enclosed cages. After insect removal, the inoculated rice plants were grown under the same greenhouse conditions. At approximately 4 weeks post‐inoculation (wpi), when viral symptoms were evident in most plants, seedlings were harvested for phenotypic documentation and molecular analyses, including gene expression and protein quantification.

### 5.4 Iodine–Potassium Iodide (I_2_‐KI) Staining of Pollen Viability

During the heading stage, rice florets predicted to bloom the following day were collected from the upper, middle, and lower portions of the panicle and placed into centrifuge tubes containing 75% ethanol. Anthers protruding more than two‐thirds of the lemma/palea length were considered mature. For staining, one anther per floret was transferred to a microscope slide and treated with 1–2 drops of 1% I_2_‐KI solution. The anthers were gently disrupted using forceps to release the pollen grains, then covered with a coverslip and lightly pressed. After standing for 2–3 min, the samples were observed under a light microscope at 10× magnification. One random field of view per slide was examined to assess pollen viability.

### 5.5 Yeast Two‐Hybrid Assay

Recombinant plasmids were transformed into the S*accharomyces cerevisiae* strain Y2HGold using the LiAc/polyethylene glycol method according to the manufacturer's protocol (Clontech, CA, USA). Positive transformants were selected on SD/‐Leu/‐Trp medium and incubated at 30°C for 3 days. Individual colonies were resuspended in sterile distilled water and transferred to SD/‐Ade/‐His/‐Leu/‐Trp medium for 3 days at 30°C to assess interaction‐dependent growth. Each bait (BD) or prey (AD) construct was co‐transformed with an empty vector to test for autoactivation. Controls included yeast strains co‐expressing an AD‐fused protein of interest and an empty BD vector (and vice versa). Positive and negative controls were AD‐T + BD‐53 and AD‐T + BD‐Lam, respectively, as previously described [44].

### 5.6 Bimolecular Fluorescence Complementation (BiFC) Analysis

Full‐length coding sequences of *RLCK22*, *MADS1*, *MADS15*, and *P3IP1* were cloned into BiFC vectors pFGC‐nYFP or pFGC‐cYFP [70] to generate in‐frame fusions with the N‐terminal (YFP^N^) or C‐terminal (YFP^C^) fragments of YFP. The constructs were verified by sequencing and introduced into *Agrobacterium tumefaciens* strain GV3101. Agroinfiltration of *N. benthamiana* leaves was performed using standard protocols [44]. YFP fluorescence was imaged at 48 h post‐infiltration (hpi) using a confocal laser scanning microscope (Nikon AX‐R, Nikon, Tokyo, Japan). Primers used for cloning are listed in Table S3.

### 5.7 Co‐Immunoprecipitation (Co‐IP) Assay

The coding sequences of *MADS1* and *MADS15* were fused to a 3×Myc tag at the C‐terminus, *RLCK22* to a 3×HA or GFP tag at the C‐terminus, and P3IP1 to GFP at the N‐terminus. All constructs were cloned into the *pCAMBIA2300* binary vector under the control of the CaMV 35S promoter. The constructs were transformed into *Agrobacterium tumefaciens* strain GV3101 and infiltrated into *N. benthamiana* leaves. The agrobacterial cultures were mixed according to the combinations shown in the indicated Figures and adjusted to OD_600_ = 1.0 prior to infiltration. Leaf tissue (0.5 g) was collected 48 hpi and homogenized in 2 mL lysis buffer [25 mm Tris‐HCl (pH 7.5), 150 mm NaCl, 10 mm DTT, 1 mm EDTA, 10% glycerol, 2% Triton X‐100, and 1× EDTA‐free protease inhibitor cocktail (Roche)]. The lysate was incubated on ice for 30 min, centrifuged at 12 000 × g for 20 min at 4°C (twice), and immunoprecipitated using indicated monoclonal antibodies. For in vivo Co‐IP in rice, Myc‐tagged *RLCK22* transgenic lines were used. Polyclonal antibodies against rice MADS1 and MADS15 were custom‐generated by immunizing rabbits with gene‐specific peptide antigens. Immune complexes were washed three times and analyzed by Western blot using anti‐Myc, anti‐HA, or anti‐GFP antibodies as indicated.

### 5.8 Luciferase Complementation Imaging (LCI) Assay

LCI assays were carried out as previously described [45]. Full‐length coding sequences of *RLCK22*, *P3IP1*, and GFP were individually fused to the N‐ or C‐terminal halves of the firefly luciferase (LUC) gene, generating the constructs RLCK22‐NLuc, GFP‐NLuc, CLuc‐GFP, and CLuc‐P3IP1. The constructs were transformed into *Agrobacterium tumefaciens* strain GV3101. Equal volumes of Agrobacterium cultures containing different combinations were mixed to a final OD_600_ of 1.0 and co‐infiltrated into four separate sites on the same *N. benthamiana* leaf. Two days post‐infiltration (dpi), 0.2 mm luciferin (Promega, Madison, WI, USA) was infiltrated into the same sites 30 min prior to imaging. Luminescence signals were detected using a NightSHADE LB 985 imaging system (Berthold Technologies, Germany).

### 5.9 Protein Expression and Purification

The coding sequences of *MADS1*, *MADS15*, and *P3IP1* were cloned into different expression vectors for the production of tag‐fusion proteins: pMAL‐c2X (MBP‐tag, New England Biolabs) for MBP‐RLCK22, and pGEX‐6P (GST‐tag, GE Healthcare) for GST‐P3IP1, GST‐RLCK22, GST‐MADS1, and GST‐MADS15. All constructs were transformed into *Escherichia coli* strain BL21 (DE3; TransGen Biotech, Beijing, China) for protein expression. Fusion proteins were purified using glutathione Sepharose 4B (GE Healthcare) for GST‐tagged proteins, and amylose resin (NEB) for MBP‐tagged proteins, following the manufacturers’ protocols.

### 5.10 Cell‐Free Protein Degradation Assay

Total cell‐free extracts were prepared from 30‐day‐old WT, P3IP1‐Myc‐overexpressing, and *rlck22* mutant rice plants using protein extraction buffer containing 50 mm Tris‐HCl (pH 8.0), 0.5 m sucrose, 1 mm MgCl_2_, 10 mm EDTA (pH 8.0), and 5 mm DTT. Purified recombinant GST‐RLCK22 protein was incubated with the extracts in the presence of 1 mm ATP, with or without 50 µm MG132, at 4°C. The reactions were terminated at indicated time points by adding SDS loading buffer. Protein degradation was assessed by immunoblotting using indicated antibodies.

### 5.11 In Vitro Phosphorylation Assay

GST‐ and MBP‐tagged recombinant proteins were expressed in *Escherichia coli* and purified as described in the Protein Expression and Purification section. In vitro phosphorylation reactions were performed in a 50 µL reaction mixture containing 20 mm HEPES (pH 7.5), 10 mm MgCl_2_, 5 mm CaCl_2_, 1 mm DTT, and 0.1 mm ATP. GST‐MADS1, GST‐MADS15, and GST‐P3IP1 were individually incubated with MBP‐RLCK22 at 30°C for 30 min. The reactions were terminated by adding 4× SDS loading buffer and heating at 95°C for 5 min. Phosphorylation of the proteins was detected by Western blotting with phospho‐specific antibodies.

### 5.12 In Vitro Ubiquitination Assay

The in vitro ubiquitination assay was performed as previously described [45]. The 50 µL reaction mixture contained crude wheat E1 enzyme extract (GenBank accession: GI:136632), 40 µg of *Arabidopsis thaliana* UBC10, 10 µg of MBP‐P3IP1, 10 µg of GST‐RLCK22, and 2 mg of purified ubiquitin (UBQ14; At4g02890), all supplemented in ubiquitination buffer. Reactions were incubated at 30°C for 1 h and terminated by adding 4× SDS loading buffer, followed by heating at 95°C for 10 min. Ubiquitination was analyzed by Western blot using anti‐ubiquitin or substrate‐specific antibodies.

### 5.13 RNA Isolation and Quantitative Real‐Time PCR Analysis

Total RNA was extracted from rice seedlings using TRIzol reagent (Takara, Japan) following the manufacturer's instructions. First‐strand cDNA synthesis was performed using First‐Strand Synthesis Master Mix (Lablead, Beijing). Quantitative real‐time PCR (RT‐qPCR) was carried out using 2× RealStar Fast SYBR qPCR Mix (GenStar, Beijing, China) on a real‐time PCR system, following the manufacturer's protocol. The expression levels of target genes were normalized to that of *OsEF1α*, and relative gene expression was calculated using the 2^−ΔΔCt^ method. The sequences of primers used for RT‐qPCR are listed in Table S3.

### 5.14 RNA Sequencing and Data Analysis

Healthy and RGSV‐infected WT, *mads1*, and *mads15* rice plants were used for RNA sequencing analysis. Leaf sheaths were collected from each genotype, immediately frozen in liquid nitrogen, and stored at −80°C until use. Each biological replicate consisted of pooled tissues from 10 individual plants, with three biological replicates per treatment.

Total RNA was extracted using the FastPure Plant Total RNA Isolation Kit (Vazyme, China) following the manufacturer's instructions. RNA quality was evaluated by agarose gel electrophoresis, Nanodrop spectrophotometry, and Agilent 2100 Bioanalyzer analysis. High‐quality RNA samples were used for library preparation and transcriptome sequencing on the Illumina platform.

Raw sequencing reads were processed to remove adapter sequences and low‐quality bases using Sickle and SeqPrep. Clean reads were aligned to the *Oryza sativa* reference genome (MSU Rice Genome Annotation Project, Release 7) using TopHat2. The mapped reads were assembled into transcripts using StringTie and annotated by comparison with the MSU7 annotation database. Transcript abundance was quantified in fragments per kilobase of transcript per million mapped reads (FPKM) using RSEM. Differentially expressed genes (DEGs) were identified using DESeq2, with adjusted p‐value (false discovery rate, FDR) ≤ 0.05 and |log_2_ fold change| ≥ 1 as thresholds.

### 5.15 Statistical Analysis

Data analysis was performed using IBM SPSS Statistics version R24.0.0.2 software. For pairwise comparisons, a two‐tailed Student's *t*‐test was used. For comparisons among multiple groups, one‐way analysis of variance (ANOVA) followed by Tukey's multiple comparison test was performed. A significance threshold of α = 0.05 was applied. The number of biological replicates (*n*) for each experiment is specified in the corresponding figure legends. Densitometric quantification of immunoblot band intensities was conducted using ImageJ software (National Institutes of Health, Bethesda, MD, USA).

## Author Contributions

S.Z. and J.W. designed the experiments. Y.W., F.W., S.H., D.Y., X.L., X.Z., and N.L. performed the experiments; Y.W., P.G., C.L., J.Z., S.Z., and J.W. analyzed the data; Y.W., S.Z., and J.W. wrote the manuscript; M.W., Q.J., Y.W., S.Z., and J.W. revised the manuscript; All authors worked collaboratively to edit and revise the paper.

## Conflicts of Interest

The authors declare no conflicts of interest.

## Supporting information




**Supporting File 1**: advs75503‐sup‐0001‐SuppMat.docx.

**Supporting File 2**: advs75503‐sup‐0002‐TableS1.xlsx.

**Supporting File 3**: advs75503‐sup‐0003‐TableS2.xlsx.

**Supporting file 4**: advs75503‐sup‐0004‐TableS3.xlsx

## Data Availability

The data that support the findings of this study are available in the supplementary material of this article.
