# Supplementary material for: A Virus‐Inducible E3–RLCK–MADS Module Coordinates Suppression of Plant Immunity and Fertility in Rice
Source: Adv Sci (Weinh). 2026 May 3;13(41):e75503. doi: 10.1002/advs.75503 (PMC13335636; doi:10.1002/advs.75503)
Supplement: Supplementary file 1 — Supporting File 1: advs75503‐sup‐0001‐SuppMat.docx. [file ADVS-13-e75503-s003.docx]

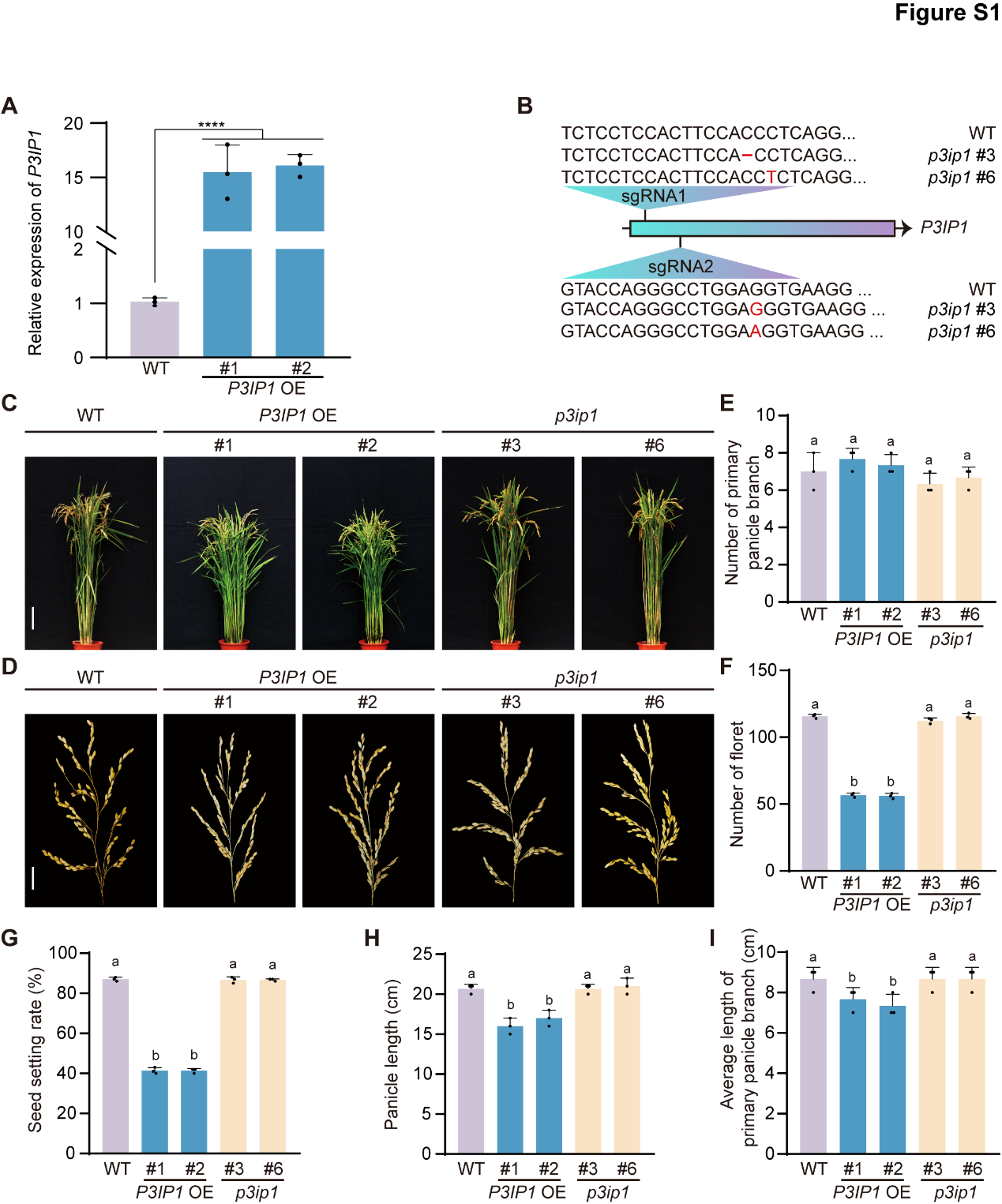


**Figure S1. Phenotypic and molecular characterization of *P3IP1* transgenic rice lines.**

A) RT-qPCR of *P3IP1* OE lines. *OsEF1α* was used as an internal reference (*n* = 3).

B) Genotyping validation of *P3IP1* CRISPR/Cas9 knockout lines. Two mutant lines (#3 and #6) were selected for phenotypic analysis.

C) Morphology of WT, *P3IP1* OE, and *p3ip1* transgenic rice lines. Bar = 15 cm.

D) Morphology of panicle from WT, *P3IP1* OE, and *p3ip1* transgenic rice lines. Bar = 5 cm.

E) Statistical analysis of primary panicle branch number in WT, *P3IP1* OE, and *p3ip1* transgenic rice lines (*n* = 3).

F) Statistical analysis of floret number in WT, *P3IP1* OE, and *p3ip1* transgenic rice lines (*n* = 3).

G) Statistical analysis of seed setting rate in WT, *P3IP1* OE, and *p3ip1* transgenic rice lines (*n* = 3).

H) Statistical analysis of panicle length in WT, *P3IP1* OE, and *p3ip1* transgenic rice lines (*n* = 3).

I) Statistical analysis of average length of primary panicle branch in WT, *P3IP1* OE, and *p3ip1* transgenic rice lines (*n* = 3).

Data were presented as mean ± *SD*. Data in (A) were analyzed using Student’s *t*-test (*****P* < 0.0001). Diﬀerent lowercase letters above bars (E-I) indicate signiﬁcant diﬀerences (*P* < 0.05) based on a one-way ANOVA test.


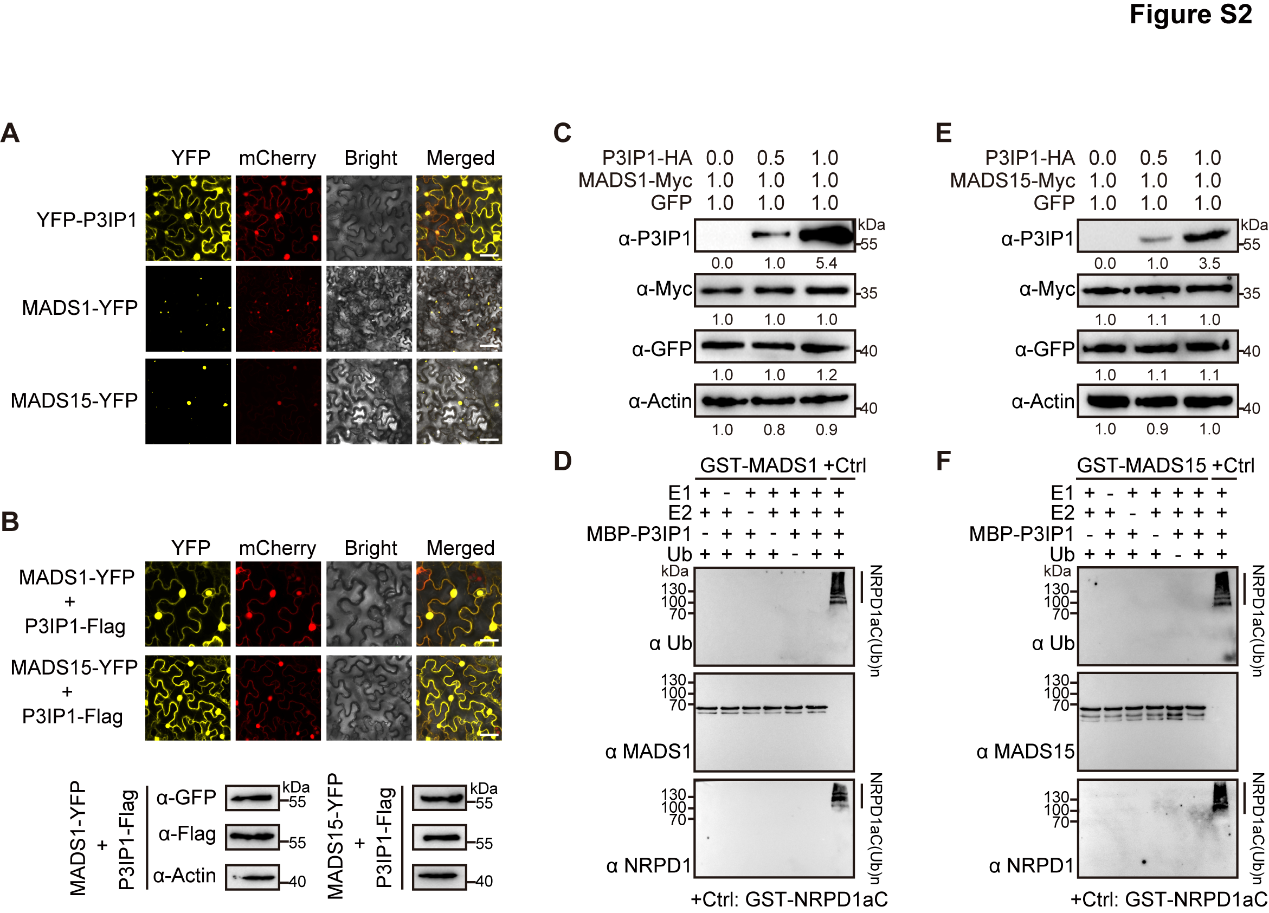


**Figure S2. Analysis of P3IP1 effects on the subcellular localization, stability, and ubiquitination of MADS1 and MADS15.**

A) Subcellular localization of P3IP1, MADS1 and MADS15 expressed individually in *N. benthamiana*. mCherry was used as a nuclear localization marker. Bar = 50 μm.

B) Subcellular localization of MADS1-YFP or MADS15-YFP co-expressed with P3IP1-Flag in *N. benthamiana*. mCherry was used as a nuclear localization marker. Bar = 50 μm.

C) and E) Transient co-expression of P3IP1-HA with MADS1-Myc C) or MADS15-Myc E) in *N. benthamiana* leaves at increasing amounts of *Agrobacterium* carrying P3IP1-HA. Actin was used as internal control.

D) and F) *In vitro* ubiquitination assays to test whether P3IP1 directly ubiquitinates MADS1 D) or MADS15 F).


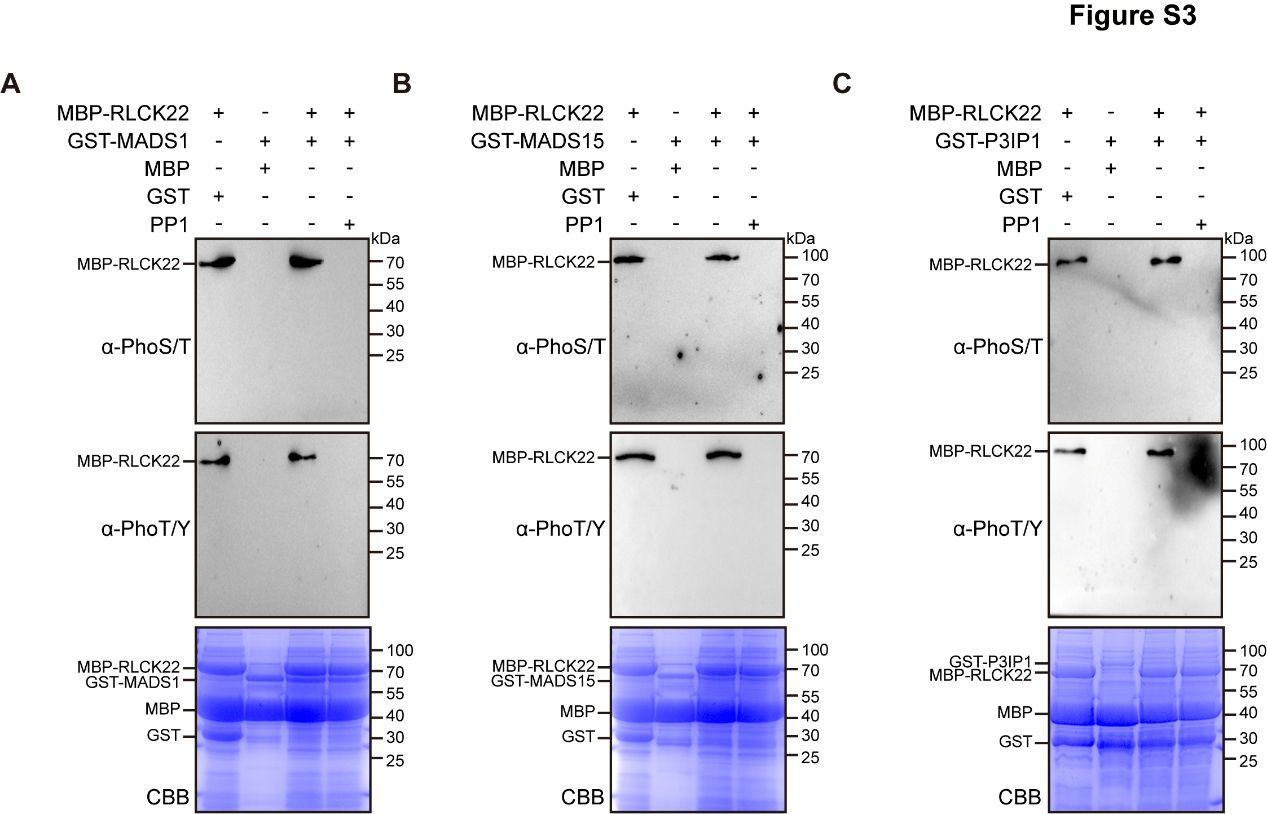


**Figure S3. *In vitro* kinase assays examining RLCK22 autophosphorylation and substrate phosphorylation.**

*In vitro* kinase assays were performed using recombinant RLCK22 protein. Phosphorylation was detected by anti-phospho-Thr/Tyr antibodies to test RLCK22 autophosphorylation activity and whether RLCK22 can phosphorylate MADS1 A), MADS15 B), and P3IP1 C). PPI, Protein phosphatase inhibitor.

**
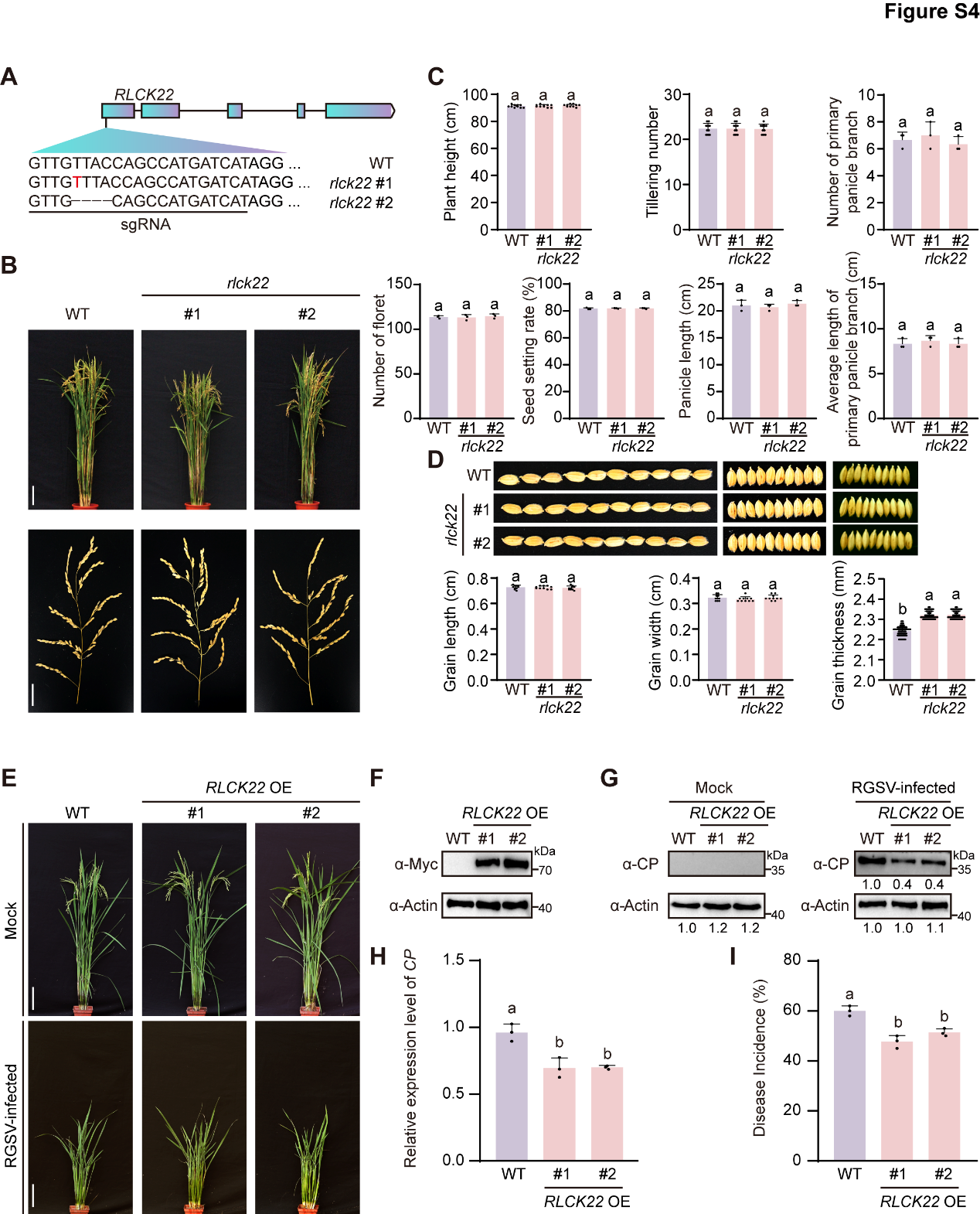
**

**Figure S4. Phenotypic analysis of *rlck22* mutants.**

A) Sequence alignment showing mutations in *rlck22* CRISPR mutant lines #1 and #2.

B) Plant and panicle morphology of WT and *rlck22* mutants at the heading stage. Upper panel, bar = 15 cm. Lower panel, bar = 5 cm.

C) Statistical analysis of plant height (*n* = 10), tillering number (*n* = 10), primary panicle branch number (*n* = 3), floret number (*n* = 3), seed setting rate (*n* = 3), panicle length (*n* = 3) and average length of primary panicle branch (*n* = 3) of WT and *rlck22* mutant rice plants.

D) Morphology and statistical analysis of grain length (*n* = 10), width (*n* = 10) and thickness (*n* = 100) from WT and *rlck22* mutant rice plants.

E) Phenotypic comparison of WT and two independent *RLCK22* OE lines (#1, #2) upon mock-inoculation or RGSV infection at 4 wpi. Bar = 10 cm.

F) Immunoblot analysis of *RLCK22* OE lines. Actin was used as internal control.

G) Immunoblot analysis of RGSV CP in WT and *RLCK22* OE plants at 4 wpi. Actin was used as internal control.

H) RT-qPCR quantification of *CP* transcripts in WT and *RLCK22* OE plants at 4 wpi. Expression normalized to *OsEF1α* (*n* = 3).

I) Disease incidence of WT and *RLCK22* OE plants infected with RGSV (*n* = 3).

Data were presented as mean ± *SD*. Diﬀerent lowercase letters above bars indicate signiﬁcant diﬀerences (*P* < 0.05) based on a one-way ANOVA test.


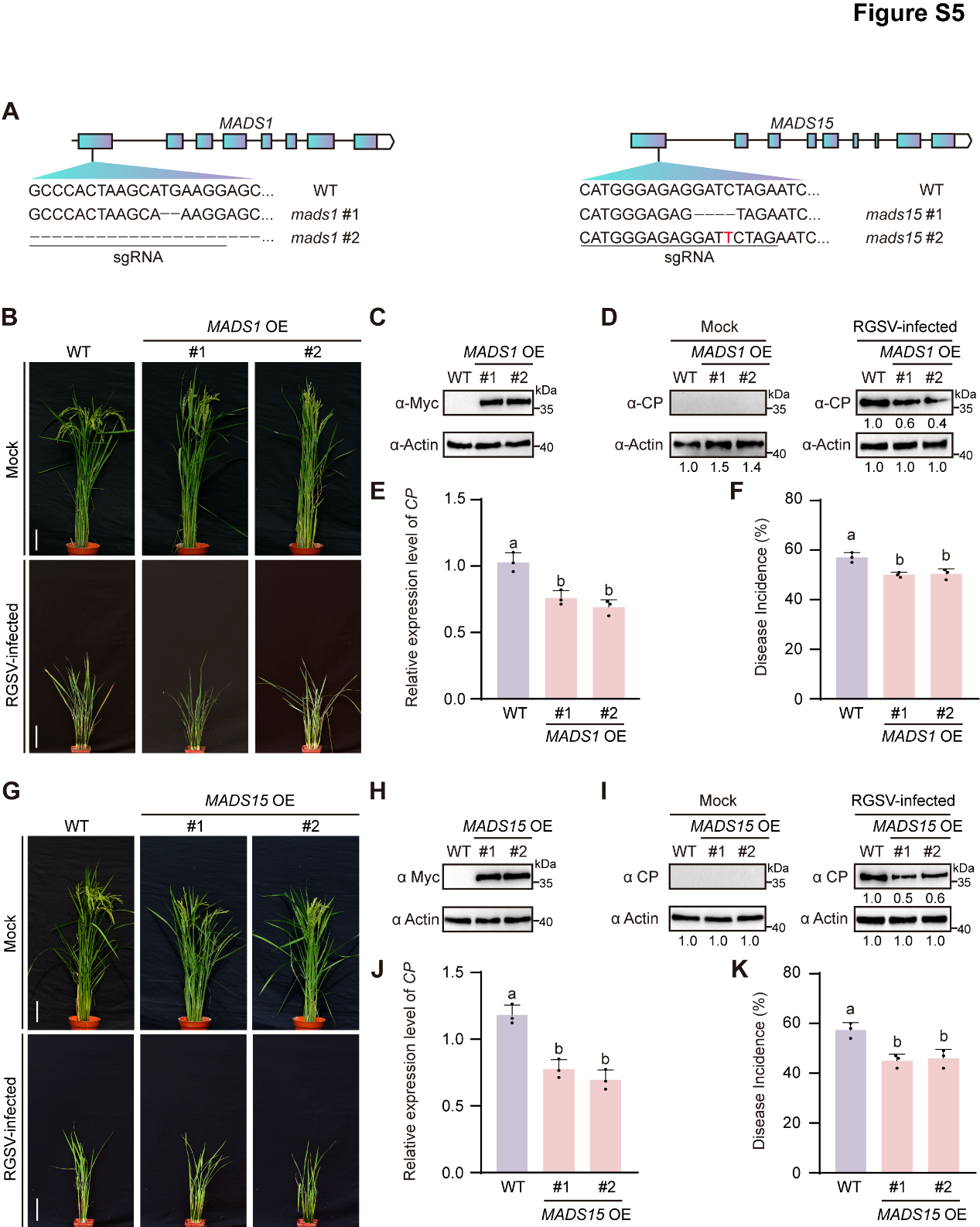


**Figure S5. Antiviral characterization of *MADS1* and *MADS15* transgenic rice plants.**

A) Genotyping validation of *mads1 and mads15* CRISPR/Cas9 knockout lines.

B) and G) Phenotypic comparison of WT and two independent *MADS1* B) or *MADS15* G) OE lines (#1, #2) upon mock-inoculation or RGSV infection at 4 wpi. Bar = 10 cm.

C) and H) Immunoblot analysis of *MADS1* C) or *MADS15* H) OE lines. Actin was used as internal control.

D) and I) Immunoblot analysis of RGSV CP in WT and *MADS1* D) or *MADS15* I) OE plants at 4 wpi. Actin was used as internal control.

E) and J) RT-qPCR quantification of *CP* transcripts in WT and *MADS1* E) or *MADS15* J) OE plants at 4 wpi. Expression normalized to *OsEF1α* (*n* = 3).

F) and K) Disease incidence of WT and *MADS1* F) or *MADS15* K) OE plants infected with RGSV (*n* = 3).

Data were presented as mean ± *SD*. Diﬀerent lowercase letters above bars indicate signiﬁcant diﬀerences (*P* < 0.05) based on a one-way ANOVA test.


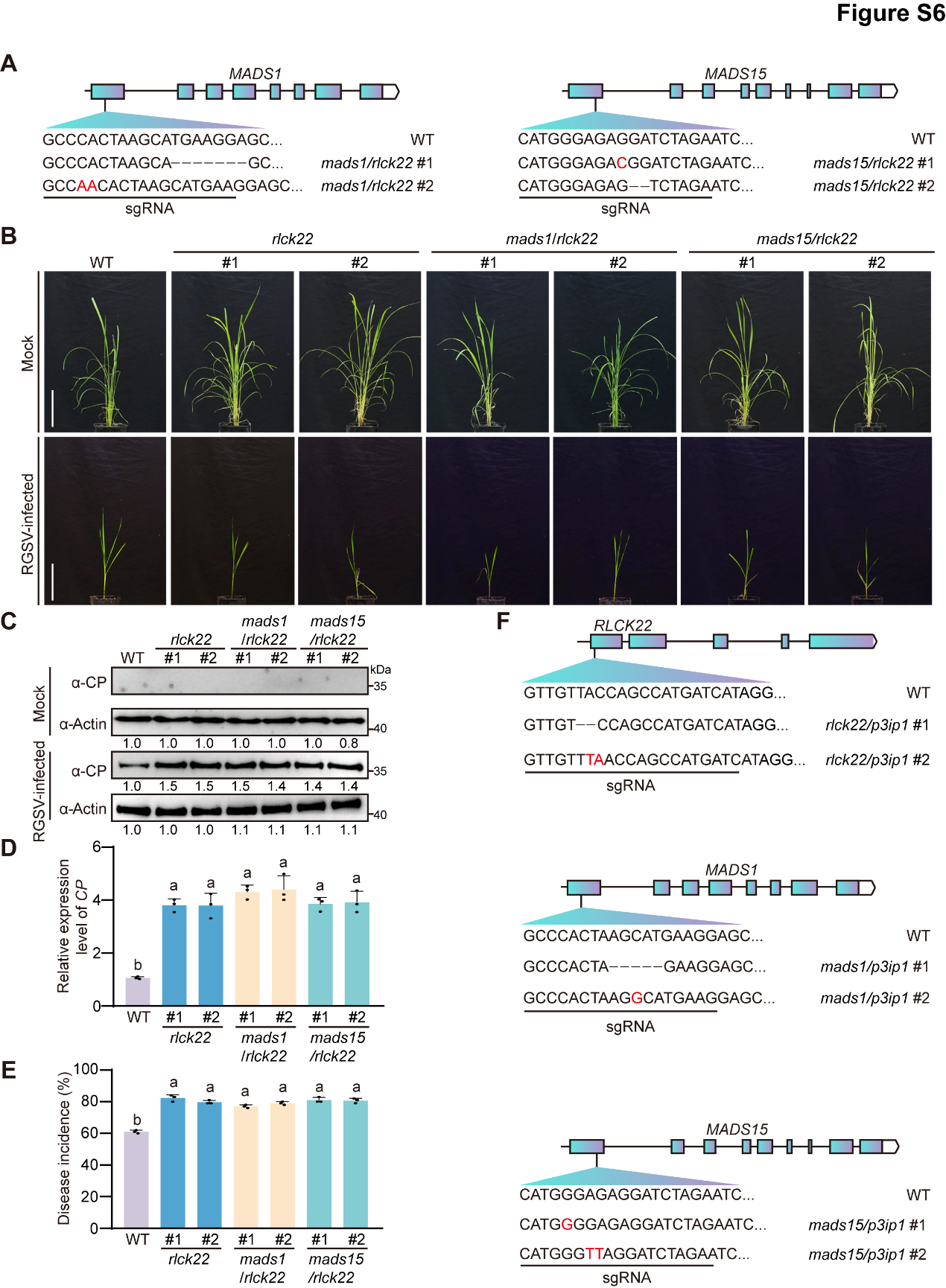


**Figure S6. Antiviral phenotypic analysis of *mads1*/*rlck22* and *mads15*/*rlck22* double mutant rice plants.**

A) Genotyping validation of *mads1/rlck22* and *mads15/rlck22* double knockout lines.

B) Phenotypic comparison of WT and mutant rice plants upon mock-inoculation or RGSV infection at 4 wpi. Bar = 10 cm.

C) Immunoblot analysis of RGSV CP in WT and mutant rice plants at 4 wpi. Actin was used as internal control.

D) RT-qPCR quantification of *CP* transcripts in WT and mutant rice plants at 4 wpi. Expression normalized to *OsEF1α* (*n* = 3).

E) Disease incidence of WT and mutant rice plants infected with RGSV (*n* = 3).

F) Genotyping validation of *rlck22/p3ip1*, *mads1/p3ip1* and *mads15/p3ip1* double knockout lines.

Data were presented as mean ± *SD*. Diﬀerent lowercase letters above bars indicate signiﬁcant diﬀerences (*P* < 0.05) based on a one-way ANOVA test.


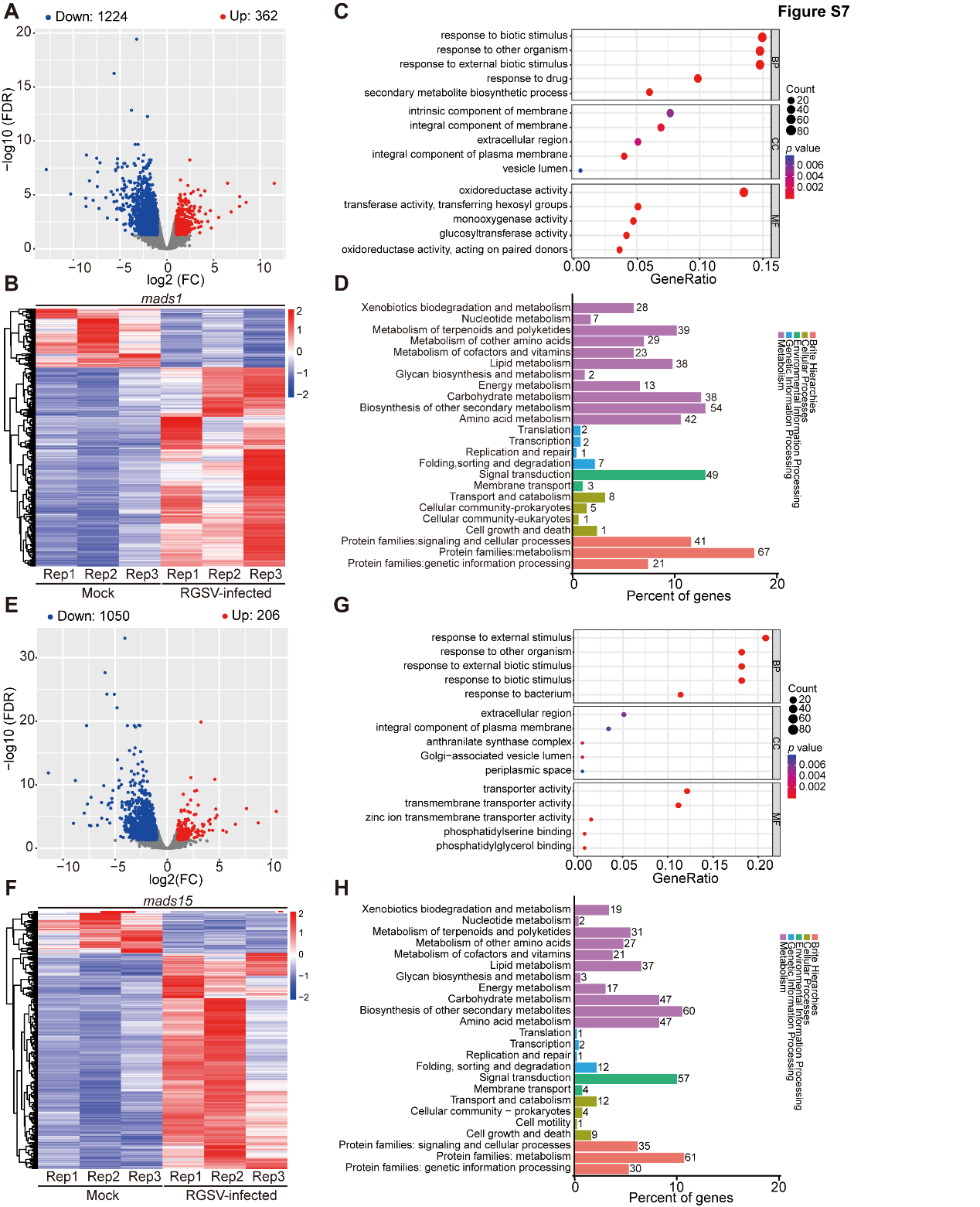


**Figure S7. Transcriptome analysis of *mads1* and *mads15* mutants upon RGSV infection.**

A) and E) Volcano plot showing DEGs between mock-inoculated and RGSV-infected *mads1* mutant plants.

B) and F) Heatmap of gene expression profiles from three biological replicates.

C) and G) GO enrichment analysis of DEGs categorized by biological process (BP), cellular component (CC), and molecular function (MF) terms.

D) and H) KEGG pathway enrichment analysis of DEGs.


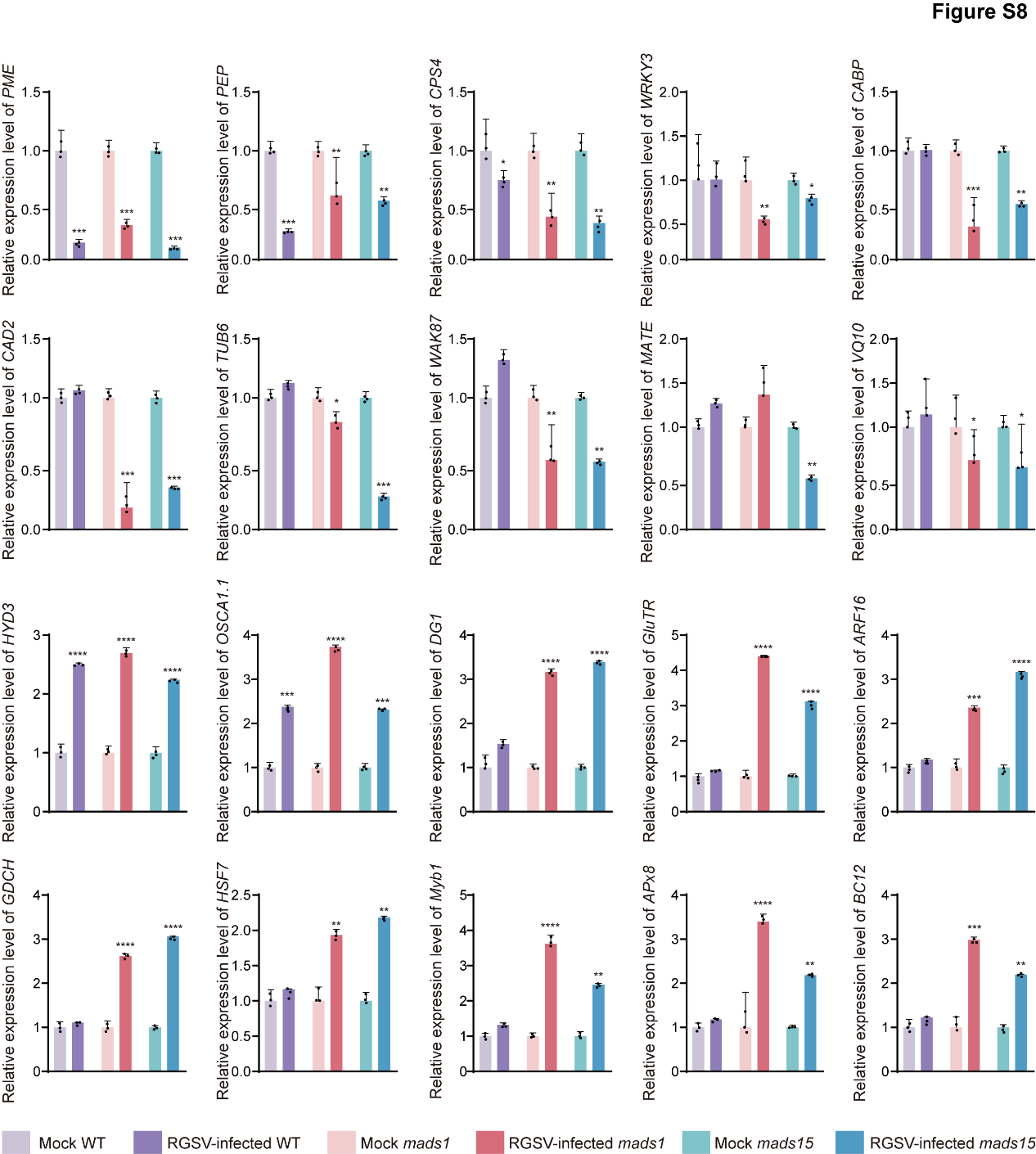


**Figure S8. RT-qPCR validation of selected DEGs identified in *mads1* and *mads15* mutants.**

Expression levels of 20 selected DEGs were measured in WT, *mads1*, and *mads15* plants upon mock-inoculation and RGSV infection. *OsEF1α* was used as an internal reference. Data were presented as mean ± *SD* (*n* = 3). (**P* < 0.05, ***P* < 0.01, ****P* < 0.001, *****P* < 0.0001) were based on Student’s *t*-test.
